# Genetic regulation of the development of mating projections in *Candida albicans*

**DOI:** 10.1080/22221751.2020.1729067

**Published:** 2020-02-21

**Authors:** Weihong Liang, Guobo Guan, Chao Li, Clarissa J. Nobile, Li Tao, Guanghua Huang

**Affiliations:** a Department of infectious diseases, Huashan Hospital, Fudan University, Shanghai, People’s Republic of China; b State Key Laboratory of Genetic Engineering, School of Life Sciences, Fudan University, Shanghai, People’s Republic of China; c State Key Laboratory of Mycology, Institute of Microbiology, Chinese Academy of Sciences, Beijing, People’s Republic of China; d Department of Molecular and Cell Biology, University of California, Merced, CA, USA

**Keywords:** Candida albicans, mating projection, sexual reproduction, cAMP/PKA signalling pathway, Cbk1, RAM pathway, MAPK pathway

## Abstract

*Candida albicans* is a major human fungal pathogen, capable of switching among a range of morphological types, such as the yeast form, including white and opaque cell types and the GUT (gastrointestinally induced transition) cell type, the filamentous form, including hyphal and pseudohyphal cell types, and chlamydospores. This ability is associated with its commensal and pathogenic life styles. In response to pheromone, *C. albicans* cells are able to form long mating projections resembling filaments. This filamentous morphology is required for efficient sexual mating. In the current study, we report the genetic regulatory mechanisms controlling the development of mating projections in *C. albicans*. Ectopic expression of *MTL*α1 in “**a**” cells induces the secretion of α-pheromone and promotes the development of mating projections. Using this inducible system, we reveal that members of the pheromone-sensing pathway (including the pheromone receptor), the Ste11-Hst7-Cek1/2 mediated MAPK signalling cascade, and the RAM pathway are essential for the development of mating projections. However, the cAMP/PKA signalling pathway and a number of key regulators of filamentous growth such as Hgc1, Efg1, Flo8, Tec1, Ume6, and Rfg1 are not required for mating projection formation. Therefore, despite the phenotypic similarities between filaments and mating projections in *C. albicans*, distinct mechanisms are involved in the regulation of these two morphologies.

## Introduction

**Figure 1.**
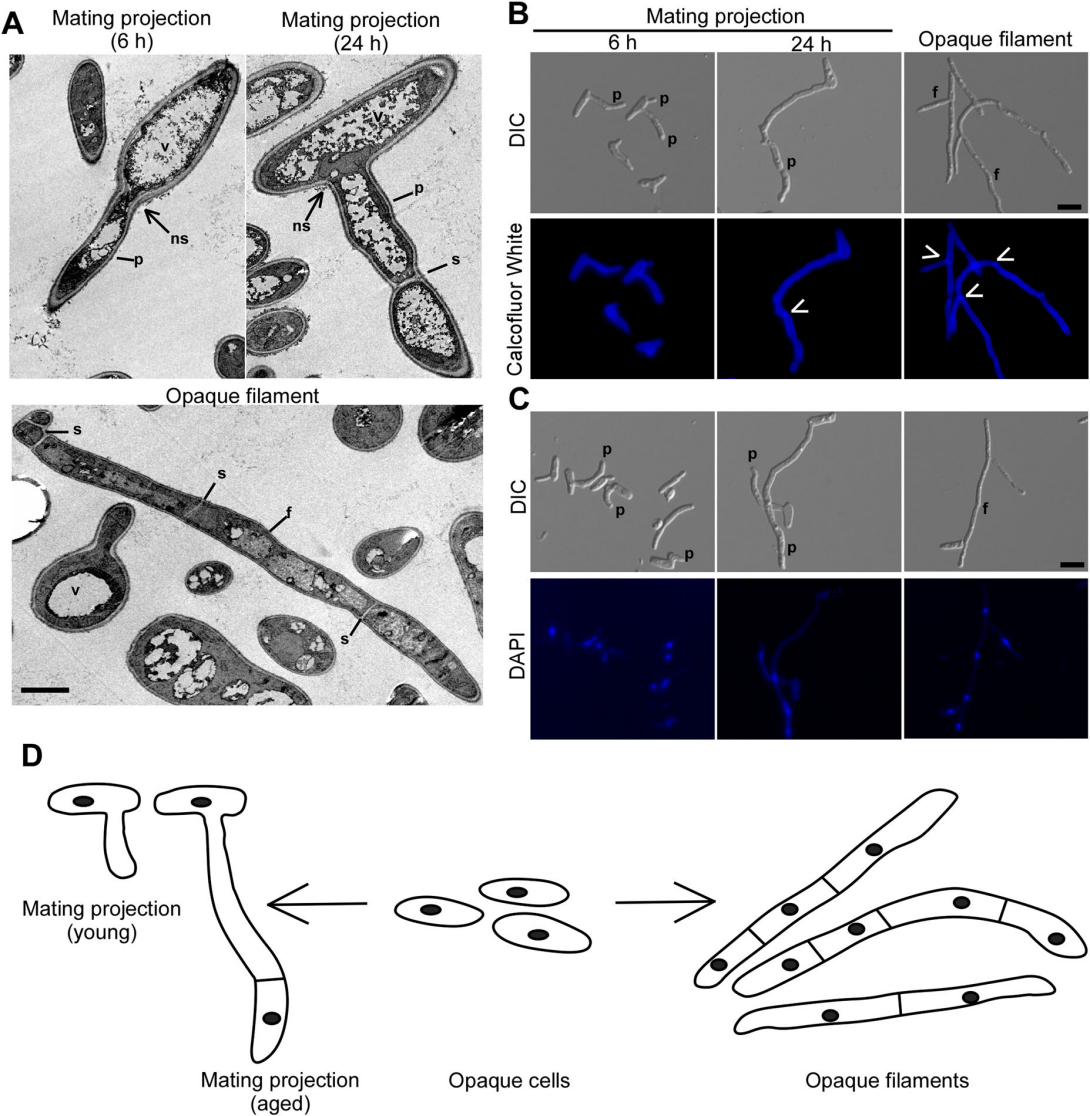
Morphologies of mating projections and opaque filaments of *C. albicans*. To induce mating projections, opaque cells (1 × 10^7^ cells/mL) were incubated in liquid Lee’s medium and treated with 50 μM α-factor at 25°C for 6 or 24 h. To induce filaments, opaque cells were incubated in SOR medium overnight at 25°C. (A) Transmission electron microscope (TEM) images. Scale bar, 2 µm. p, projection; f, filamentation; v, vacuole; s, septum; ns, no septum. (B and C) Calcofluor White and DAPI staining assays. Scale bar, 10 µm. White arrows, septum. p, projection; f, filamentation. (D) Diagrams of opaque cells, mating projections, and opaque filaments.

Sexual reproduction is pervasive among fungi and is associated with genetic diversification, evolution of antifungal resistance and new traits, and adaptation to environmental changes [1–4]. Although the major processes and regulatory signalling pathways are generally conserved among different fungal species, the strategies used for sexual reproduction are highly diversified [5,6]. The major human fungal pathogen *Candida albicans* and the model yeast *Saccharomyces cerevisiae* diverged from a common ancestor approximately 300 million years ago [7]. Both species are able to undergo sexual or parasexual reproduction under certain conditions. Although the overall mating responses are similar in the two yeast species, there are several species-specific features of this conserved biological process. For example, in order to mate, *C. albicans* must first undergo a morphological transition, called white-opaque switching, to become mating-competent [8]. White cells of *C. albicans* are round and small and mating-incompetent, whereas opaque cells are elongated and large and mate approximately one million times more efficiently than white cells [8,9]. This *C. albicans* white-opaque transition provides an additional regulatory mechanism for controlling sexual reproduction and could be beneficial for adapting to environmental changes. Another unique mating response characteristic of *C. albicans* and its closely related species is that opaque cells form long mating projections in response to sexual pheromone, while cells of *S. cerevisiae* form short polarized “shmoo” morphologies (Figure 1) [10]. Formation of filamentous-like projections functions as an additional regulatory process of mating and could be another adaptive behaviour of *C. albicans* to its natural niches. We previously demonstrated that mating projections facilitate invasive growth of *C. albicans* in a mouse skin infection model [11].

The regulatory control of filamentous growth in *C. albicans* has been extensively investigated over the past two decades [12–14]. Mating projections of *C. albicans* are morphologically similar to, but functionally distinct from, filaments. Despite the importance of mating projections in the life history of *C. albicans*, the regulatory mechanisms controlling mating projection formation are largely unknown. In this study, we set out to determine the genetic regulatory mechanisms of mating projection development in *C. albicans*. We demonstrate that the pheromone-response Ste11-Hst7-Cek1/2-mediated MAPK signalling cascade and the RAM pathway are required for the development of mating projections. To our surprise, the cAMP-PKA signalling pathway and several critical regulators of filamentous growth such as Tec1, Hgc1, and Ume6 are not essential for the induction of mating projections. Our findings indicate that both conserved and distinct mechanisms are involved in the regulation of mating projection formation in *C. albicans*.

## Materials and methods

### Plasmids, strains, and media

The strains used in this study are listed in supplementary Table S1. Modified Lee’s glucose medium [15] and YPD (1% yeast extract, 2% peptone, 2% glucose) were used for routine culture of *C. albicans* strains. Sorbitol (SOR) medium (synthetic complete defined (SCD) medium supplemented with 1 M sorbitol) was used for the induction of opaque filaments as previously described [16]. To induce filaments, opaque cells (1 × 10^6^) were incubated in 1 mL liquid SOR medium at 25°C with shaking at 200 RPM for overnight growth.

The wild type strain SN152(**a**/-) was used as the control for most experiments. To construct the *MTL*α1-overexpression plasmid pNIM1-*MTL*α1, the ORF region of *MTL*α1 was amplified from the genomic DNA of *C. albicans* by PCR using primers OE*MTL*α1-F and OE*MTL*α1-R. The PCR products were digested with *Sal*I and *Bam*HI and subcloned into plasmid pNIM1. Plasmid pNIM1-*MTL*α1 was linearized by digestion with *Sac*II and *Apa*I and used to transform *C. albicans*. To generate *C. albicans MTL***a**/- or *MTL*-/α strains, one allele of the *MTL* locus was deleted using the linearized plasmid pSFS2a-*MTL*KO (L23.14) [17]. The *SAT1*/flipper cassette of the transformants was then excised by growing them on YPM medium (1% yeast extract, 2% peptone, 2% maltose).

To construct the *WOR1-*overexpression plasmid p*ACT-WOR1-SAT1,* the *SAT1* cassette was amplified from plasmid pNIM1 and inserted into the *Hind*III/*Kpn*I site of plasmid *pACT-WOR1* [18]*.* The resulting plasmid p*ACT-WOR1-SAT1* was linearized with *AscI* and transformed into the WT strain and the RAM (regulation of Ace2 and morphogenesis) pathway mutants.

To delete *HGC1* in strain BWP17**a**, plasmid pSFS2a-*HGC1*KO was linearized with *Apa*I and *Sac*I and used for transformation as described previously [19]. The fusion PCR recombination strategy [20] was used to delete *MF*α1, *STE2, UME6, TEC1, CBK1, MOB2, HYM1, KIC1, CAS4,* and *SOG2* in strain SN152 of *C. albicans*. Primers marker-F and marker-R were used to amplify the selection markers *HIS1* and *ARG4* from plasmid pGEM-HIS1, pRS-ARG4ΔSpeI [21]. To delete the first allele, cells of strain SN152 were transformed with the fusion PCR product of the *CdARG4* flanked by 5′- and 3′- flanking fragments of the corresponding gene. To delete the second allele, the resulting heterozygous mutants were transformed with fusion PCR products of the *CdHIS1* flanked by 5′- and 3′- flanking fragments of corresponding genes. Correct replacement of the target gene was verified by colony PCR. Primers used for fusion PCR assays are listed in Table S2.

### Pheromone-induced mating projection assays

To obtain the opaque phenotype, *C. albicans* cells were plated on Lee’s glucose or Lee’s GlcNAc medium at 25°C. Opaque cells from homogenous colonies were used for all mating projection assays. A 14-mer α-pheromone peptide (GFRLTNFGYFEPGK) was chemically synthesized and used for the induction of mating projections in *C. albicans* as previously described [11]. Opaque cells were cultured in liquid Lee’s glucose medium at 25°C for 36 h and then inoculated into fresh Lee’s glucose medium (1 × 10^7^ cells/mL) with or without 50 μM α-pheromone peptide. After 6 or 24 h of incubation, cells were examined under a microscope.

To induce mating projections by ectopic expression of *MTL*α1, opaque cells were transformed with plasmid pNIM1-*MTL*α1 and cultured in liquid Lee’s medium at 25°C for 36 h to stationary phase. Cells were then collected, re-inoculated, and incubated in fresh liquid Lee’s glucose medium with or without 40 μg/mL doxycycline for 24 h. To calculate the percentages of mating projections, at least 100 cells of each sample were examined.

### Microscopy assays

Cells grown in liquid Lee’s glucose or Lee’s GlcNAc medium were collected and washed with 1 x PBS. Calcofluor White was used to stain chitin septa and DAPI (4′, 6-diamidino-2-phenylindole) was used to stain nuclei as described previously [22]. Transmission electronic microscopy (TEM) assays were performed according to our previous publication [22]. Briefly, cells were fixed with 0.5% polyxymethylene and 2.5% glutaraldehyde in a buffer solution (0.2 M PIPES, piperazine-N,N′-bis-2-ethanesulfonic acid, 1 mM MgCl_2_, 1 mM CaCl_2_, 0.1 M sorbitol, pH 6.8) for two hours at 4°C. After a gentle washing, cells were dehydrated in ascending grades of acetone solutions, and then embedded in Spurr resin.

### Quantitative real-time PCR (Q-RT–PCR) assay

Q-RT–PCR assays were performed according to our previous publication [23]. Opaque or mating projection cells were grown in liquid Lee’s glucose medium at 25°C for 24 h. Cells were collected and washed with 1 x PBS. Total RNA was extracted with the GeneJET RNA Purification kit (Thermo scientific, Waltham, MA, USA). RevertAid H Minus reverse transcriptase (Thermo scientific, Waltham, MA, USA) was used to synthesize cDNA. Quantification of transcripts was performed using SYBR green (TOYOBO CO., LTD) in a Bio-Rad CFX96 real-time PCR detection system. The expression levels of each sample were normalized to that of *ACT1*.

### Mating assays

Quantitative mating assays were performed as previously described [11]. Briefly, 1 × 10^4^ opaque cells of “**a**” and “α” strains were mixed in 20 μL ddH_2_O and spotted onto Lee’s glucose medium. After 48 h of incubation at 25°C, the mating mixtures were collected and replated on the SCD medium for prototrophic selection growth. Colonies grown on the selected media were counted, and mating efficiencies were calculated as previous described [8].

## Results

### Morphologies of mating projections and opaque filaments

In response to α-factor, opaque cells of *C. albicans* were able to develop long mating projections (Figure 1). The morphology of mating projections is generally similar to that of opaque filaments induced by sorbitol (SOR) medium [16]. To distinguish mating projections and opaque filaments, we performed transmission electron microscopy (TEM) assays and observed that mating projections had large vacuoles and irregular cell wall architectures (Figure 1A). Calcofluor white and DAPI staining assays demonstrated that newly developed/young mating projections (treated with α-factor for six hours) contained only a single nucleus, whereas fully-developed/aged or mature mating projections (treated with α-factor for 24 h) contained multiple nuclei. Similar to fully-developed mating projections, the opaque filaments consist of multiple cellular compartments separated by septa, and each compartment contains a single nucleus. Opaque filaments, on the other hand, were straighter than mating projections and had parallel sidewalls. To compare and contrast the two morphologies, a descriptive diagram is shown in Figure 1D.

### Ectopic expression of *MTL*α1 promotes mating projection formation in “**a**” cells

**Figure 2.**
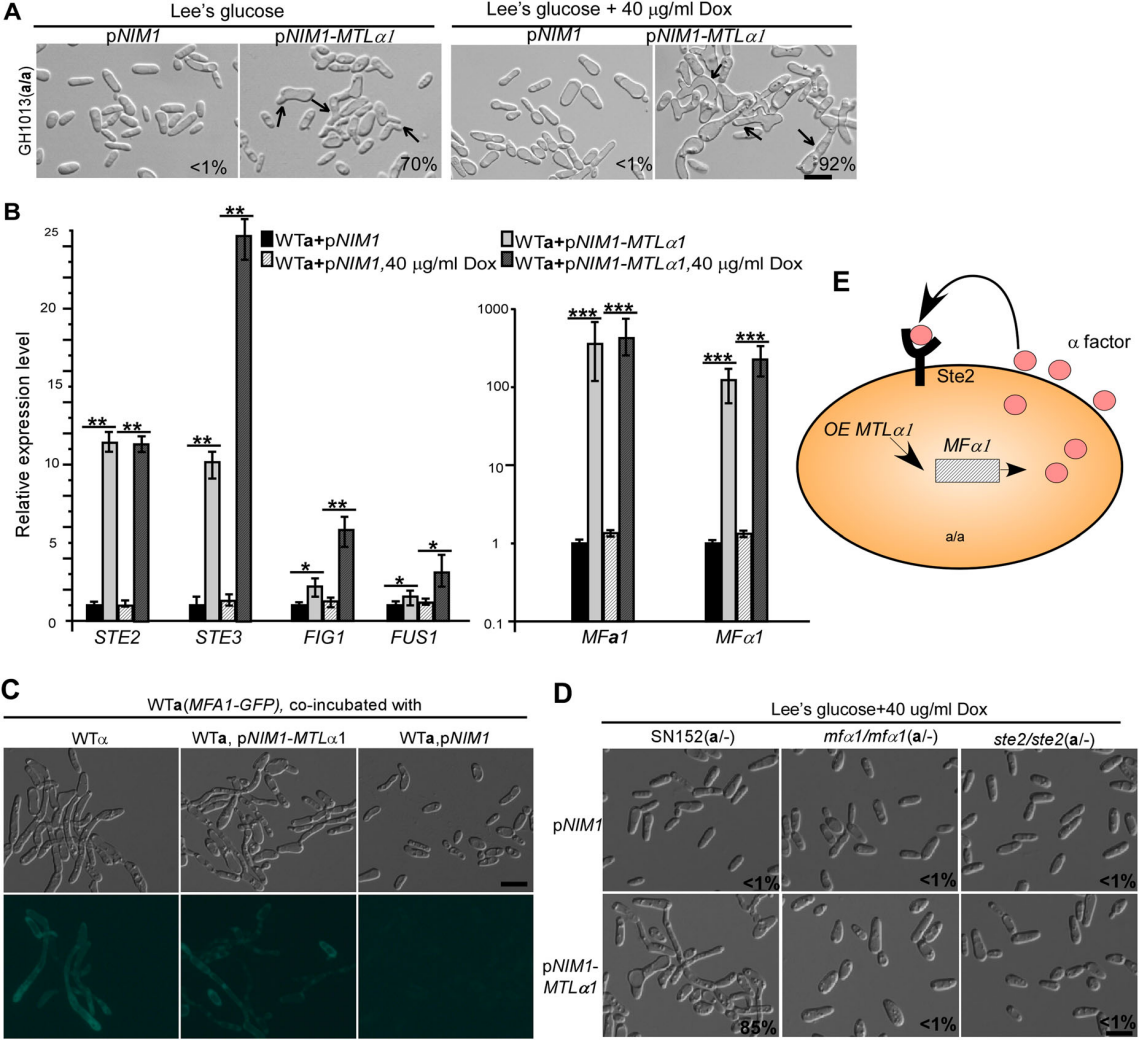
Ectopic expression of *MTL*α1 promotes mating projection formation in “**a**” cells of *C. albicans*. (A) Ectopic expression of *MTL*α1 in “**a**” cells. pNIM1, plasmid containing a doxycycline-controlled *TETon* promoter. Percentages of mating projection cells are indicated in the corresponding images. Black arrows indicate mating projections. (B) Relative expression levels of mating and pheromone-response genes in the control (WT**a** + pNIM1) and *MTL*α1-ectopically expressing strains (WT**a** + pNIM1-*MTL*α1) in Lee’s glucose medium with or without 40 μg/mL doxycycline (Dox). Error bar, standard deviation (SD). *P < 0.05, **P < 0.01 (Student’s *t*-tests, two tailed). (C) Ectopic expression of *MTL*α1 in “**a**” cells induces the secretion of α-factor. An *MFA1p-GFP* reported strain served as a reporter for detecting the secretion of α-factor. Scale bar, 10 µm. (D) MFα1 and its receptor Ste2 are required for the development of mating projection induced by ectopic expression of *MTL*α1. Scale bar, 10 µm. Diagram of *MTL*α1-induced mating projection formation in “**a**” cells. Ectopic expression of *MTL*α1 in “**a**” cells induces the secretion of α-factor. α-Factor then binds to its receptor Ste2 and activates the mating response signalling pathway and mating projection formation through an auto-feedback mechanism.

The mating type locus of *C. albicans* encodes four transcriptional regulators: **a**1, **a**2, α1, and α2 [24]. Mtl**a**1 and Mtlα2 are homeodomain proteins and form a heterodimer that represses the transcription of “**a**” or “α” cell-specific genes, while Mtl**a**2 and Mtlα1 function as transcriptional activators of “**a**” and “α” cell-specific genes, respectively [24,25]. Ectopic expression of *MTL***a**2 in “α” cells of *C. albicans* induces the development of mating projections [26]. We ectopically expressed *MTL*α1 in “**a**” cells under the control of the *TETon* promoter. As shown in Figure 2A, ectopic expression of *MTL*α1 efficiently induced the formation of mating projections in “**a**” cells in the presence of 40 μg/mL doxycycline. Even in the absence of doxycycline, the introduction of a *TETon*-promoter-controlled *MTL*α1 in “**a**” cells, also promoted the development of mating projections, implying that low levels of *MTL*α1 expression due to “leaky” transcription is enough to activate the development of mating projections. This leaky expression could be due to the fact that the opaque-specific *OP4* minimal promoter is a component of the *TETon* promoter. This efficient induction system provided us with a convenient assay to explore the genetic regulatory mechanisms of the development of mating projections.

### Ectopic expression of *MTL*α1 in “**a**” cells induces the expression of mating-specific genes

We predicted that the induction of mating projections in *MTL*α1-ectopically expressing cells could be due to the secretion of α-factor and the self-activation of the mating-response pathway in “**a**” cells. We first tested the relative expression levels of *MFA1* (encoding the **a**-factor precursor), *MFα1* (encoding the α-factor precursor), *STE2* (encoding the receptor for α-factor [27]), *STE3* (encoding the receptor for **a**-factor), and *FIG1* and *FUS1* (encoding mating-required membrane proteins) in *MTL*α1-ectopically expressing cells. As shown in Figure 2B, the expression of these mating-associated genes was significantly upregulated in *MTL*α1-ectopically expressing cells even in the absence of doxycycline.

To verify the secretion of α-factor in *MTL*α1-ectopically expressing “**a**” cells, a reporter “**a**” strain (GH1600) carrying an *MFA1* promoter-controlled GFP cassette was co-cultured with *MTL*α1-ectopically expressing “**a**” cells [11]. As shown in Figure 2C, GFP expression was observed in cells of the reporter strain co-cultured with *MTL*α1-ectopically expressing “**a**” cells, but not in cells co-cultured with “**a**” cells of the control strain carrying the empty vector. These results demonstrate that *MTL*α1-ectopically expressing “**a**” cells are able to secrete α-factor and in turn induce the development of mating projections.

To further confirm this self-activating mechanism, we next examined the effect of inactivation of *MFα1* and its receptor-encoding gene *STE2* on the development of mating projections in *MTL*α1-ectopically expressing “**a**” cells. We found that both *MFα1* and *STE2* were essential for the development of mating projections (Figure 2D), indicating that this self-activating mechanism controls mating projection formation in *MTL*α1-ectopically expressing “**a**” cells (Figure 2E).

### The Ste11-Hst7-Cek1/2-mediated mitogen-activated protein kinase (MAPK) pathway is essential for the development of mating projections

**Figure 3.**
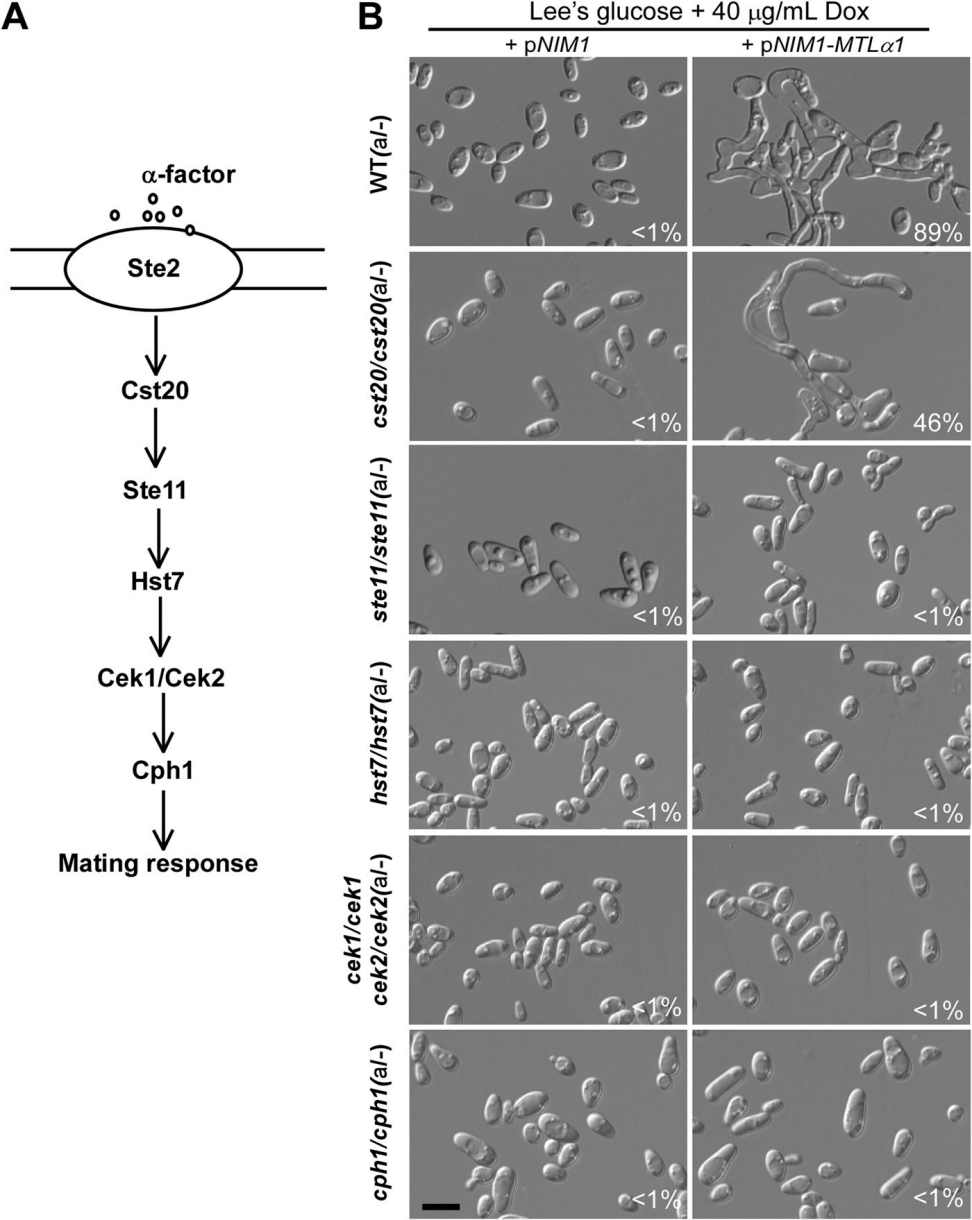
Development of mating projections in the mutants of the mating response signalling pathway. (A) Schematic diagram of the MAPK-mediated mating response signalling pathway in *C. albicans*. (B) Ectopic expression of *MTL*α1 in the mutants of the mating response signalling pathway. Opaque cells of *C. albicans* (1 × 10^7^ cells/mL) were cultured in liquid Lee’s medium containing 40 μg doxycycline at 25°C for 24 h. Percentages of mating projection cells are indicated in the corresponding images. Scale bar, 10 µm.

The conserved Ste11-Hst7-Cek1/2-mediated MAPK pathway is required for pheromone sensing, mating, and white cell filamentation in *C. albicans* [16,28–30] (Figure 3A). Therefore, it was reasonable to predict that this signalling pathway is required for mating projection formation. To test this hypothesis, we ectopically expressed *MTL*α1 in the *cst20/cst20*, *ste11/ste11*, *hst7/hst7*, and *cek1/cek1 cek2/cek2* double mutants of the MAPK pathway and its downstream transcription factor Cph1 mutant (*cph1/cph1*). As shown in Figure 3B, deletion of the MAPKKK kinase-encoding gene *CST20* attenuated but did not block the development of mating projections in the *MTL*α1-ectopically expressing strain. However, deletion of *STE11*, *HST7*, both *CEK1* and *CEK2, or CPH1* completely blocked *MTL*α1-induced mating projection development.

To verify the role of the MAPK pathway in mating projection formation, we treated opaque cells of the *ste2/ste2*, *ste11/ste11*, and *hst7/hst7* mutants with 50 μM α-factor for six hours. As expected, no mating projections were formed in these mutants (Figure S1). Consistently, deletion of *STE2* also blocked mating in *C. albicans* (Table 1). Taken together, our results indicate that the MAPK pathway is essential for mating projection formation in *C. albicans*.

**Table 1.** Mating efficiencies of the null mutants of *C. albicans*.

Cross	Mating efficiency
SN152 (**a**/-) × GH1352 (α/α)	(7.2 ± 0.8) × 10^−2^
*tpk1-/-* (**a**/-) × *tpk1-/-* (α/α)	(5.1 ± 0.3) × 10^−2^
*tpk2-/-* (**a**/-) × *tpk2-/-* (α/α)	(9.0 ± 0.4) × 10^−2^
*tpk1-/-, tpk2-/-* (**a**/-) × *tpk1-/-, tpk2-/-* (α/α)	(2.1 ± 0.1) × 10^−1^
*ste2-/-* (**a**/-) × *ste2-/-* (α/α)	<1 × 10^−9^
*hgc1-/-* (**a**/-) × *hgc1-/-* (α/α)	(3.7 ± 0.6) × 10^−2^
*ume6-/-* (**a**/-) × *ume6-/-* (α/α)	(5.5 ± 0.8) × 10^−2^
*tec1-/-* (**a**/-) × *tec1-/- (*α/α*)*	(2.3 ± 0.3) × 10^−2^
SN152 p*ACT-WOR1* (**a**/-) × SN152 p*ACT-WOR1* (α/-)	(2.6 ± 0.7) × 10^−2^
*cbk1-/-* p*ACT-WOR1* (**a**/-) × *cbk1-/-* p*ACT-WOR1* (α/-)	(1.5 ± 0.5) × 10^−3^

Notes: Opaque “a” cells (1 × 10^4^) were mixed with opaque “α” cells (1 × 10^4^) in 20 μL ddH_2_O and spotted on Lee’s medium at 25°C for 48 h. The mixtures were resuspended and cultured on SCD media for prototrophic selection growth. Colonies grown on the selected media were counted and mating efficiencies were calculated.

### The Ras1 GTPase and cAMP signalling pathway is not required for the development of mating projections

**Figure 4.**
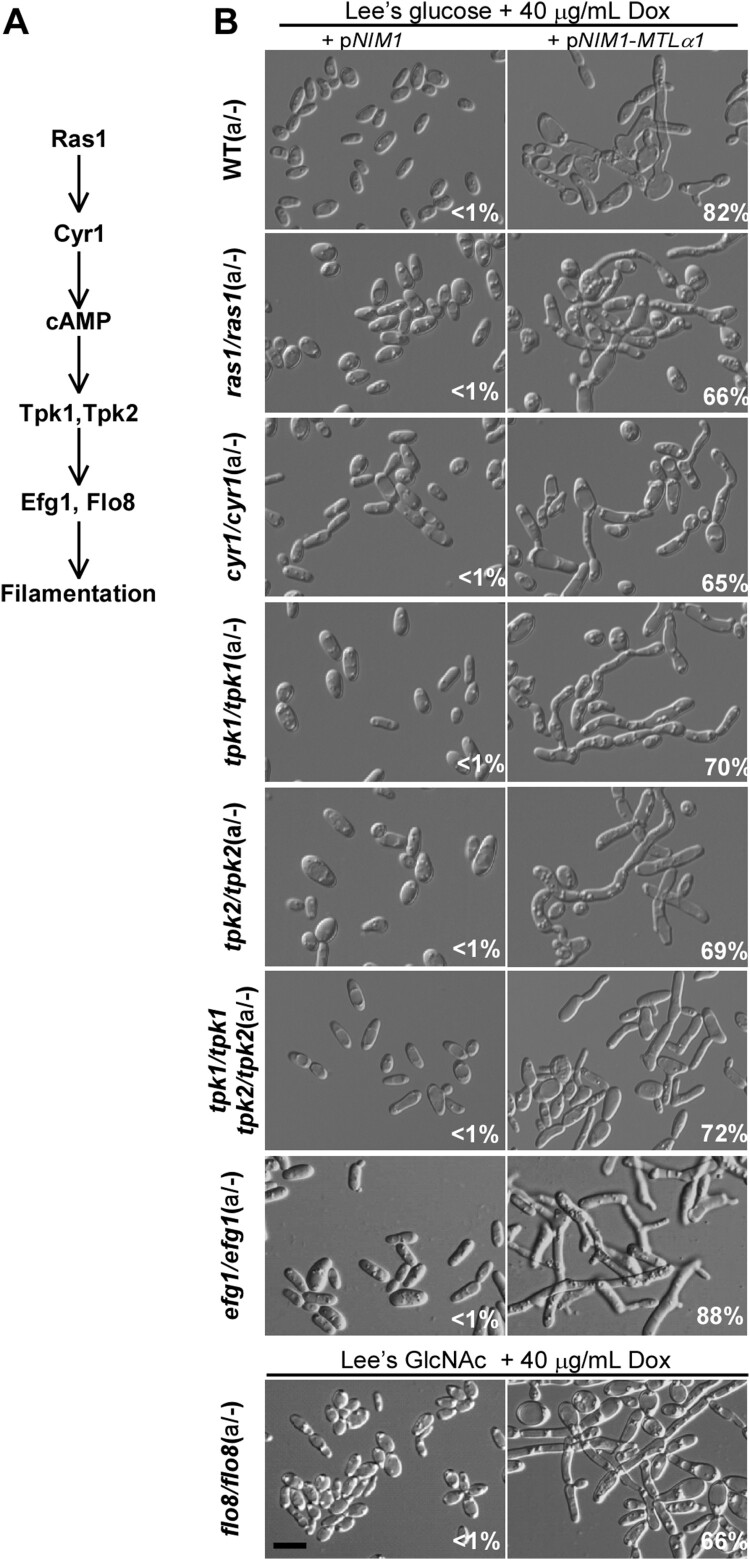
Role of the Ras-cAMP/PKA signalling pathway in the development of mating projections. (A) Diagram of the Ras-cAMP/PKA signalling pathway in *C. albicans*. (B) Ectopic expression of *MTL*α1 in the mutants of the Ras-cAMP/PKA signalling pathway. Opaque cells of *C. albicans* (1 × 10^7^ cells/mL) were cultured in liquid Lee’s glucose or Lee’s GlcNAc (only for the *flo8/flo8* mutant) medium containing 40 μg/mL doxycycline at 25°C for 24 h. Percentages of mating projection cells are indicated in the corresponding images. Scale bar, 10 µm.

Ras1 is a conserved GTPase regulating both the Ste11-Hst7-Cek1/2-mediated MAPK as well as cAMP/PKA signalling pathways in *C. albicans* [14] (Figure 4A). Although Ras1 plays a critical role in filamentous growth [31,32], we found that deletion of *RAS1* did not block *MTL*α1-induced mating projection development (Figure 4B). Cyr1 and the PKA kinase catalytic subunit play critical roles in filamentous growth in both white and opaque cells under a range of culture conditions. *CYR1* encodes the single adenylyl cyclase, and *TPK1* and *TPK2* encode two isoforms of the PKA catalytic subunit [14,16,17]. To our surprise, the *cyr1/cyr1*, *tpk1/tpk1*, and *tpk2/tpk2* single mutants, and the *tpk1/tpk1 tpk2/tpk2* double mutant were able to form mating projections when ectopically expressed with *MTL*α1 (Figure 4B). Efg1 and Flo8 are the two transcription factors downstream of the cAMP/PKA signalling pathway that play critical roles in filamentation [33]. Consistently, neither Efg1 nor Flo8 were required for *MTL*α1-induced mating projection formation (Figure 4B). We note that opaque cells of the *flo8/flo8* mutant are not stable in glucose-containing medium [34], and therefore, the induction assay for mating projection formation was performed in Lee’s GlcNAc medium for this strain.

To verify the role of the cAMP/PKA signalling pathway in mating projection formation, we treated opaque cells of the *ras1/ras1*, *cyr1/cyr1*, *tpk1/tpk1*, *tpk2/tpk2*, *tpk1/tpk1 tpk2/tpk2 double, efg1/efg1, and flo8/flo8* mutants with 50 μM α-factor for six hours. To maintain the opaque phenotype, we introduced an *ACT1* promoter-controlled *WOR1* cassette into the *flo8/flo8* mutant, generating an opaque-locked strain. As expected, cells of all of these mutants were able to form mating projections in the presence of α-factor (Figure S2). These results suggest that the Ras1 and the cAMP/PKA signalling pathways are not required for mating projection formation in *C. albicans*. Consistent with these results, inactivation of *TPK1* or *TPK2* did not reduce mating efficiency, while inactivation of both genes (in the *tpk1/tpk1 tpk2/tpk2* double mutant) even led to an increase in mating efficiency (Table 1). The results of the *TPK1* and *TPK2* mutants are consistent with our previous publication [35]. There could be crosstalk between the cAMP/PKA signalling pathway and the Ste11-Cst7-Cek1/2 mediated MAPK signalling cascade in *C. albicans* and other fungal species [35]. Inactivation of both isoforms of the PKA kinase, leading to an increase of mating efficiency, could enhance specificity of the mating response.

### Roles of key regulators of filamentous growth and the RAM pathway in mating projection development

Since the morphology of mating projections in *C. albicans* is generally similar to that of filaments and a number of filamentous-specific genes are also upregulated in mating projections [36], we next evaluated the roles of a range of positive and negative regulators of filamentation in the regulation of mating projection development. The general transcriptional repressors Tup1 and Nrg1 function as negative regulators in filamentous growth of both white and opaque cells [16,17,37]. The Rfg1 transcription factor represses filamentation in white cells [16,38], whereas Bcr1 functions as a strong repressor of filamentation in opaque cell [17]. We overexpressed *TUP1, NRG1*, *RFG1*, and *BCR1* using the *ACT1* promoter in *MTL*α1-ecotopically expressed cells and found that all overexpressing strains were able to form comparable mating projections to the control strain (not shown), suggesting that these transcriptional repressors play minor roles in the regulation of mating projection development.

**Figure 5.**
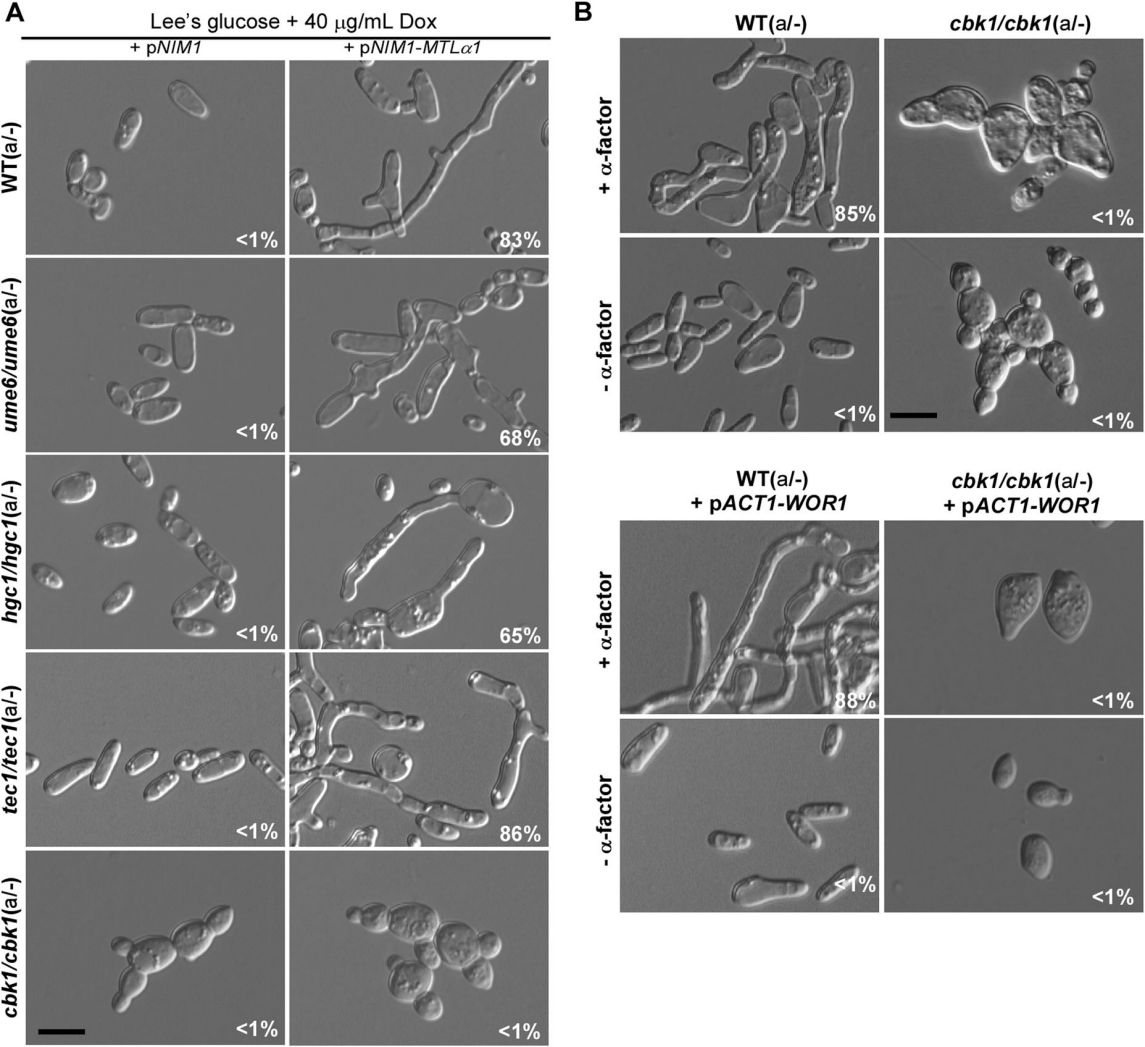
Roles of Ume6, Hgc1, Tec1, and Cbk1 in the regulation of the formation of mating projection. (A) Ectopic expression of *MTL*α1 in the *MTL***a**/- mutants of *ume6/ume6*, *hgc1/hgc1*, *tec1/tec1*, and *cbk1/cbk1*. Opaque cells of *C. albicans* (1 × 10^7^ cells/mL) were cultured in liquid Lee’s glucose medium containing 40 μg/mL doxycycline at 25°C for 24 h. Scale bar, 10 µm. (B) Treatment of the *cbk1/cbk1* and *cbk1/cbk1 *+ pACT-WOR1 strains with 50 μM α-factor. The *cbk1/cbk1 *+ pACT-WOR1 strain is a *WOR1*-overexpressing (opaque-locked) strain. Opaque cells of the two strains were treated with α-factor and incubated at 25°C for 24 h. Scale bar, 10 µm. Percentages of mating projection cells are indicated in the corresponding images.

Ume6, Tec1, Hgc1, and Cbk1 are well characterized key regulators of filamentation in *C. albicans* [37,39–43]. Ume6 is a zinc-finger DNA-binding motif transcription factor and is required for hyphal extension in *C. albicans* [40]. Tec1 is a conserved TEA/ATTS transcription factor and is downstream of the Ste11-Hst7-Cek1/2-mediated MAPK pathway in *C. albicans* and *S. cerevisiae* [39,44–46]. Tec1 is also required for pheromone response and pheromone-induced biofilm development in white cells of *C. albicans* [46]. Hgc1 is a hypha-specific G1 cyclin-related protein that is transcriptionally regulated by Efg1 and Flo8 [33,41]. Cbk1 is a conserved serine/threonine kinase of the RAM signalling network and is involved in the regulation of polarized growth and filamentation in *C. albicans* [47,48]. To examine the roles of these key regulators of filamentous growth in mating projection formation, we ectopically expressed *MTL*α1 in these mutants. As shown in Figure 5A, the *ume6/ume6, tec1/tec1,* and *hgc1/hgc1* mutants were able to form mating projections*,* but the *cbk1/cbk1* mutant exhibited a serious defect. As expected, α-factor treatment also induced the development of mating projections in the *ume6/ume6, tec1/tec1,* and *hgc1/hgc1* mutants, but not in the *cbk1/cbk1* mutant (Figure 5B and Figure S3). However, cells of the *cbk1/cbk1* mutant produced short “shmoos” in response to pheromone. Since the morphology of opaque cells of the *cbk1/cbk1* mutant was different from that of typical opaque cells of the WT, we overexpressed *WOR1* in the *cbk1/cbk1* mutant to generate an opaque-locked strain and to eliminate the effect of phenotypic switching on pheromone response. Consistently, the opaque-locked *cbk1/cbk1* mutant showed a similar defect in mating projection formation (Figure 5B).

Since the development of mating projections is important for efficient mating in *C. albicans*, we next examined the mating efficiencies of these mutants of key filamentation-related genes. As shown in Table 1, crosses of the mutants of *ume6/ume6, tec1/tec1,* and *hgc1/hgc1* showed comparable mating efficiencies to that of the WT controls. However, deletion of *CBK1* resulted in a significant reduction in mating efficiency in *C. albicans*, suggesting that the Cbk1 kinase could regulate mating efficiency via effects on the development of mating projections.

**Figure 6.**
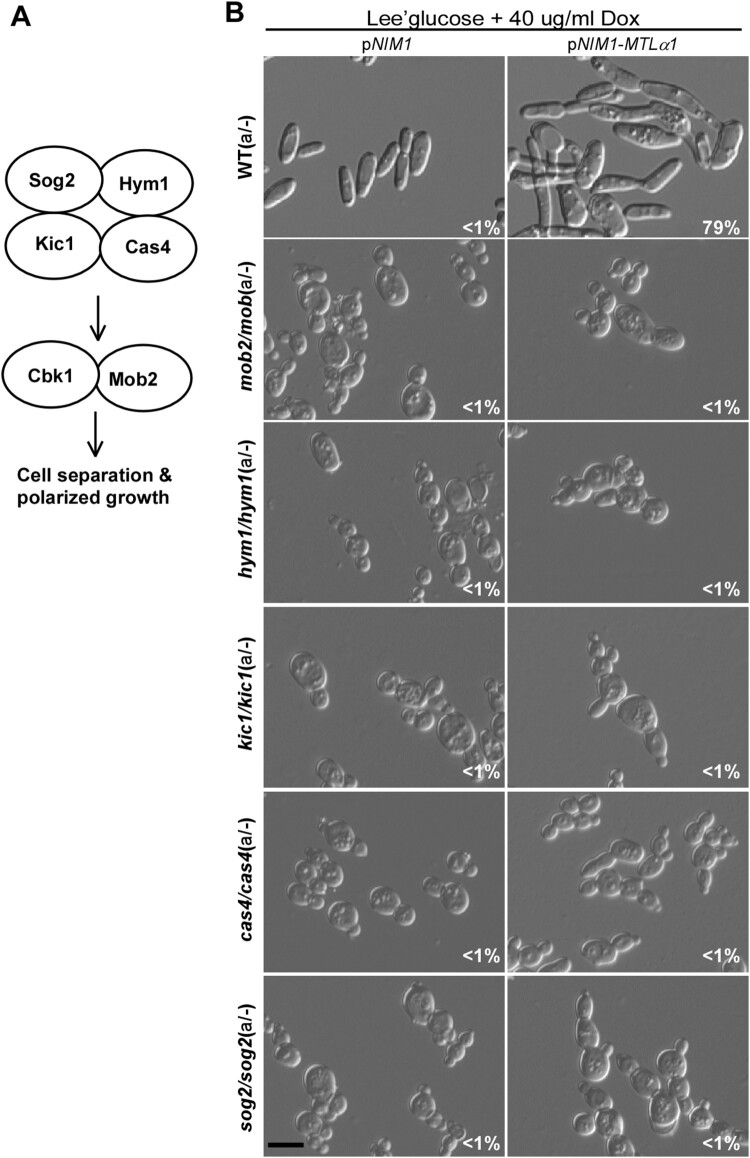
Role of the RAM pathway in the development of mating projections. (A) Diagram of the RAM pathway in *C. albicans*. (B) Ectopic expression of *MTL*α1 in the RAM pathway mutants. Opaque cells of the mutants (1 × 10^7^ cells/mL) were cultured in Lee’s glucose medium containing 40 μg/mL doxycycline at 25°C for 24 h. Scale bar, 10 µm.

Cbk1 is a key member of the RAM pathway in fungi [47]. We next examined the role of the other members of the RAM pathway in the regulation of mating projections in *C. albicans* (Figure 6A). We generated mutants of *SOG2, HYM1*, *KIC1*, *CAS4*, and *MOB2* genes in *C. albicans*. To induce the formation of mating projections, we ectopically expressed *MTL*α1 in these mutants and found that inactivation of any gene of this pathway resulted in a serious defect in mating projection formation (Figure 6B).

To verify these results, we treated opaque-locked cells of the *sog2/sog2, hym1/hym1, kic1/kic1, cas4/cas4*, and *mob2/mob2* mutants (containing an *ACT1* promoter-controlled *WOR1* cassette) with 50 μM α-factor for six hours. As expected, cells of all of these mutants failed to form mating projections in the presence of α-factor (Figure S4).

To establish a link between the mating response and RAM pathways, we performed quantitative RT–PCR assays (Figure S5). Inactivation of the RAM pathway genes (*CAS4, HYM1, KIC1, MOB2*, and *SOG2*) but not *CBK1* decreased the expression levels of mating-related genes. However, deletion of genes of the RAM pathway had no obvious effects on the expression of polarity-related genes (*CDC42*, *CDC24*, and *BEM3*). Also, inactivation of genes of the MAPK pathway had no significant effects on the expression of the RAM pathway genes at the transcriptional level. Taken together, our results suggest that the RAM pathway plays a critical role in the regulation of mating projection development in *C. albicans*.

## Discussion

In response to pheromone, cells of *C. albicans* are capable of growing elongated morphologies called mating projections. Given that mating-competent opaque cells are the minority cell population in nature [49], these elongated cellular morphologies could facilitate *C. albicans* cells in reaching suitable mating partners. In the current study, we investigated the genetic regulatory mechanisms of mating projection development in *C. albicans*. We examined the roles of several signalling pathways in the formation of mating projections and demonstrated that the pheromone sensing signalling pathway is essential for the development of mating projections in *C. albicans* (Figure 3). However, the conserved cAMP/PKA pathway and several key regulators of filamentation including Tec1, Ume6, and Hgc1 are not required for this process (Figures 4 and S3). We also determined that the Cbk1 kinase and the RAM pathway, which are involved in the control of cellular polarization, are required for the development of mating projections (Figures 5 and S4). This finding is consistent with fact that the development of mating projections is a polarized cellular response.

The mating type loci of *C. albicans* and *S. cerevisiae* differ in several aspects [24,25]. The most important difference is that the locus of the former species carries four *MAT* transcription factor-encoding genes (**a**1, **a**2, α1, and α2), while the latter species carries only three genes (**a**1, α1, and α2) and the pseudogene (**a**2). In *C. albicans*, Mtl**a**1 and Mtlα2 form a heterodimer that represses the expression of both **a**- and α-specific gene expression and the mating-competent opaque phenotype [24,25]. In *S. cerevisiae, MAT***a**2 has become a pseudogene*.* In *C. albicans,* Mtl**a**2 and Mtlα1 function as an activator of “**a**” or “α” cell-specific genes due to a rewiring of the regulatory circuit during the long term of evolution [24,25]. Ectopic expression of Mtlα1 in *C. albicans* “**a**” cells, therefore, would activate the expression of α-specific genes including the α-factor encoding gene *MF*α1. The expression and secretion of MFα would then activate the pheromone response pathway and induce the development of mating projections in “**a**” cells in a self-activating manner (Figure 2). This efficient induction system was used to explore the genetic regulatory mechanisms controlling *C. albicans* mating projection development in the current study.

The *C. albicans* Ste11-Hst7-Cek1/2 pheromone sensing pathway plays a critical role in mating and white cell filamentation but is not required for opaque cell filamentation [16]. We demonstrate that this pathway is essential for the development of mating projections in *C. albicans* (Figure 3). Inactivation of the pheromone receptor (Ste2), Ste11, Hst7, Cek1/2, or Cph1 blocked *MTL*α1- or α-factor-induced mating projection development. However, inactivation of Cst20 only partially affected the development of mating projections.

**Figure 7.**
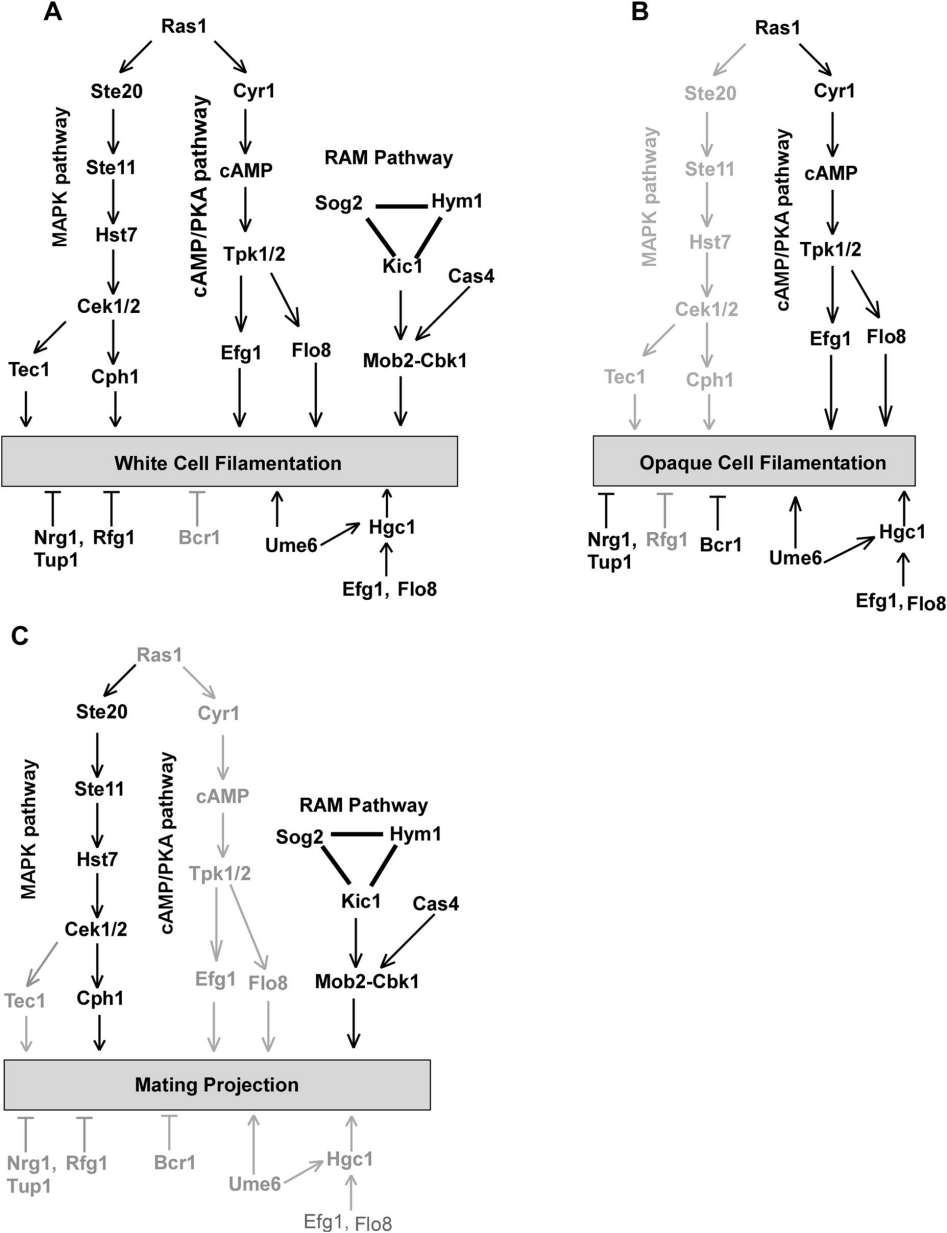
Genetic regulation of white cell filamentation (A), opaque cell filamentation (B), and mating projection formation (C) in *C. albicans*. The cAMP/PKA pathway is required for filamentous growth in both white and opaque cells, but is not required for the development of mating projections. The Ste11-Hst7-Cek1/2-mediated MAPK pathway is essential for the development of mating projections. This pathway plays a critical role in white cell filamentation but is not required for opaque cell filamentation. The RAM pathway is essential for the development of mating projections and white cell filamentation. Its role in opaque cell filamentation remains to be investigated. The Bcr1 transcription factor controls filamentous growth in opaque cells but is not involved in the regulation of mating projection formation and white cell filamentation. Ume6, Efg1, Flo8, and Hgc1 are important regulators of filamentous growth in both white and opaque cells but are not required for the development of mating projections.

The conserved cAMP/PKA pathway and its downstream regulators Efg1, Flo8, and Hgc1 are important for both white and opaque cell filamentation in *C. albicans* [16,17]. Interestingly, none of these regulators are essential for the development of mating projections. Consistently, inactivation of the cAMP/PKA pathway does not reduce mating efficiency [17]. We further found that other regulators of filamentation such as Ume6, Bcr1, Rfg1, Nrg1, and Tup1 are also not required for the development of mating projections in *C. albicans* (Figures 5 and S3). Intriguingly, the RAM pathway, which is involved in cellular polarization, is essential for the development of mating projections in *C. albicans*. Inactivation of the Cbk1 kinase completely blocked the development of mating projections and dramatically reduced mating efficiency (Figure 5 and Table 1). In *S. cerevisiae*, Cbk1 is also required for the formation of mating projections. Deletion of *CBK1* in *S. cerevisiae* leads to a defect in maintaining polarized growth of the mating projection [47], implying that the function of Cbk1 in *C. albicans* and *S. cerevisiae* is conserved. Given the pleiotropic roles of the RAM pathway in fungi, inactivation of this pathway may directly or indirectly affect the development of mating projection in *C. albicans*. Taken together, our findings suggest that the genetic regulatory mechanisms of mating projection formation are distinct from those of filamentation in both white and opaque cells of *C. albicans* (Figure 7).

The development of long mating projections in *C. albicans* and its closely related species is a unique characteristic that is critical for sexual reproduction. Although in the current study we demonstrate the roles of several signalling pathways in the regulation of mating projection development, many questions still remain to be answered. For example, why does *C. albicans* need to develop such elongated projections to mate? Is this feature associated with the development of filaments over evolutionary time? Is the development of mating projections linked to the commensal and pathogenic life styles of *C. albicans* and its closely related species? The integration of sexual reproduction with the ability to undergo morphological transitions is not unique to *C. albicans*. For example, *Cryptococccus neoformans*, a fungal pathogen that causes meningoencephalitis, forms filaments during sexual reproduction [3]. The ability to undergo morphological changes during this conserved biological process could be an adaptive behaviour for these pathogenic fungi that could be associated with virulence.

## Supplementary Material

Supplemental Material
