# Supplementary material for: Genetic regulation of the development of mating projections in Candida albicans
Source: Emerg Microbes Infect. 2020 Feb 21;9(1):413–26. doi: 10.1080/22221751.2020.1729067 (PMC7048184; doi:10.1080/22221751.2020.1729067)
Supplement: Supplemental Material [file TEMI_A_1729067_SM6265.zip › Table S1. Primers used in this study-2020.1.docx]

**Table S1. Primers used in this study**

| **Name** | **Sequence (5’ to 3’)** | **Purpose** |
| --- | --- | --- |
| OE *MTL*α1-F | CCAGCCGTCGACATGGGAAATAAAAAAAAAACTAG | Overexpression of *MTL*α1 |
| OE *MTL*α1-R | GGCGCCGGATCCTTACTTCATTATGTAAACATCCTC |  |
| RT-*STE2-*F | TACTGGTTGGTATGATGGATC | Real time PCR of *STE2* |
| RT-*STE2-*R | AAGGCAACAACAATCAATCC |  |
| RT-*STE3-*F | TGTTGGTAAGTTGGATGCTG | Real time PCR of *STE3* |
| RT-*STE3-*R | TGCATATCTTGATCCTGTCAC |  |
| RT- *FIG1-*F | AGAAGCTATGACTTGGACAGC | Real time PCR of *FIG1* |
| RT- *FIG1-*R | AGTGGTTGTTGTTGGTGTTG |  |
| RT- *FUS1-*F | TAGCAAAAGCTCTCCAAATG | Real time PCR of *FUS1* |
| RT- *FUS1-*R | TGCGATGTAGATGGTACTTTC |  |
| RT-*MFA1-*F | ATGGCTGCTCAACAACAATC | Real time PCR of *MFA1* |
| RT-*MFA1-*R | AACAGAACAAGTGGAACAGC |  |
| RT- *MF*α1*-*F | TGACAGTAACCAAGTTGTTG | Real time PCR of *MF*α1 |
| RT- *MF*α1*-*R | AGCACCAGAGGTAAGAGTAG |  |
| RT-*CBK1*-F | TGGAAGAAACTCGGTGATG | Real time PCR of *CBK*1 |
| RT-*CBK1*-R | CAACAGTGGAATACGCCATC |  |
| RT-*MOB2*-F | TCTCCTACAAAGCGTAGCAG | Real time PCR of *MOB2* |
| RT-*MOB2*-R | GGTAGTCTGTATGGAAGAACG |  |
| RT-*HYM1*-F | GATGAAGTGGAACCACAAC | Real time PCR of *HYM1* |
| RT-*HYM1*-R | CAACATCTTTCCGTGAATC |  |
| RT-*KIC1*-F | TGTTGCTGTGATTGTACGTG | Real time PCR of *KIC1* |
| RT-*KIC1*-R | CCATTGTTGTACGTTTAGTGG |  |
| RT-*CAS4*-F | CTTCAGCAGCAATTCAGTCATC | Real time PCR of *CAS4* |
| RT-*CAS4*-R | TTCTATCAGCGTGGACAGCAG |  |
| RT-*SOG2*-F | GACTCGTTGTCTTCTGTTAC | Real time PCR of *SOG2* |
| RT-*SOG2*-R | TGATGGATTCCCTGACACTTC |  |
| RT-*CDC42*-F | AATCACCCAGGAACAGGGTG | Real time PCR of *CDC42* |
| RT-*CDC42*-R | ACAGGAGGTTCTAATGCAG |  |
| RT-*CDC24*-F | GTTGGTTCTGATAGTAGTTCG | Real time PCR of *CDC24* |
| RT-*CDC24*-R | AAGATGGGTAGCAGGATCG |  |
| RT-*SEC3*-F | TGCGTGAGTCGAATGATAG | Real time PCR of *SEC3* |
| RT-*SEC3*-R | AGGTGCTTGATGAGTACCATC |  |
| RT-*BEM3*-F | GTAACATTACGCTGAAGAGT | Real time PCR of *BEM3* |
| RT-*BEM3*-R | GTTGTGATTTCGTACTTTCC |  |
| *MF*α1*-*5’flank*-*F | TTCATTTATGCACGTCAAGG | Knock out *MF*α*1* |
| *MF*α1*-*5’flank*-*R | CACGGCGCGCCTAGCAGCGGTACTATCTATCCAGTGTATGG |  |
| *MF*α1*-*3’flank*-*F | GTCAGCGGCCGCATCCCTGCAGAAGATGGAAAGCATACTG |  |
| *MF*α1*-*3’flank*-*R | AAGATAGGCAAATGCAGAAG |  |
| *MF*α1*-*Check*-*F | TACATTGTTCTGCAAGTGAC |  |
| *MF*α1*-*Check*-*R | AGTGAATCACCAGCTTATGG |  |
| *MF*α1*-*ORF*-*F | TGCCACTATTGTTGCTGCTG |  |
| *MF*α1*-*ORF*-*R | AGTCTAAAACCGGCTTCAGC |  |
| *STE2-*5’flank*-*F | ACTGGGATGATTTTGTATGG | Knock out *STE2* |
| *STE2-*5’flank*-*R | CACGGCGCGCCTAGCAGCGGAGGAATACTGGTTGTTGATCG |  |
| *STE2-*3’flank*-*F | GTCAGCGGCCGCATCCCTGCTTGGAATACAATTCCTCGTC |  |
| *STE2-*3’flank*-*R | AATGTTTCAATGCCTGTACC |  |
| *STE2-*Check*-*F | AGATGATCGACTGAACATTG |  |
| *STE2-*Check*-*R | TGCGTTATTTGTTCCAAGTG |  |
| *STE2-*ORF*-*F | ATTCCAGGATTAGATCAACC |  |
| *STE2-*ORF*-*R | TCCATTAGTTACATCGGATG |  |
| *UME6-*5’flank*-*F | GCTTTACATAATTGGTGATA |  |
| *UME6-*5’flank*-*R | CACGGCGCGCCTAGCAGCGGGTAATCATTTATACAGTGGA | Knock out *UME6* |
| *UME6-*3’flank*-*F | GTCAGCGGCCGCATCCCTGCAATTAGGCTAAGTTAAGAAT |  |
| *UME6-*3’flank*-*R | CGAGAATATTAATGTGTGCA |  |
| *UME6-*Check*-*F | AGGTTAGATATATAATTGGCT |  |
| *UME6-*Check*-*R | GTGTAAAATCAAATGGACCA |  |
| *UME6-*ORF*-*F | TCTGGAGTTGGGACTAGGAT |  |
| *UME6-*ORF*-*R | CCACTTTGTTGAGATGAAGA |  |
| *TEC1-*5’flank*-*F | TTGAATAGACTGTGGTATCA | Knock out *TEC1* |
| *TEC1-*5’flank*-*R | CACGGCGCGCCTAGCAGCGGGAGTAGCTTGCGACATCATA |  |
| *TEC1-*3’flank*-*F | GTCAGCGGCCGCATCCCTGCACTAGTGAGTTTTAGTGAAC |  |
| *TEC1-*3’flank*-*R | CACCAAATCAATTATGAACA |  |
| *TEC1-*Check*-*F | TAATACCTCACACCAACTC |  |
| *TEC1-*Check*-*R | CAAGCAATTACAACAATACA |  |
| *TEC1-*ORF*-*F | CATGATGTGTACGACCAAGG |  |
| *TEC1-*ORF*-*R | TGTTCCTCGTGACTGTTGAA |  |
| *CBK1-*5’flank*-*F | ACTCGTTCATACCCGTTTGT | Knock out *CBK1* |
| *CBK1-*5’flank*-*R | CACGGCGCGCCTAGCAGCGGGCTGATCGAAATTCATAAGA |  |
| *CBK1-*3’flank*-*F | GTCAGCGGCCGCATCCCTGCGAATGCGTTATAGTAGTGTG |  |
| *CBK1-*3’flank*-*R | TGACCACCTATTGTTGATAC |  |
| *CBK1-*Check*-*F | ATCACAGCATTTTACTACA |  |
| *CBK1-*Check*-*R | GTTGCAATTCCGGTTCTCA |  |
| *CBK1-*ORF*-*F | CTTGGATTGTTGCATTATAC |  |
| *CBK1-*ORF*-*R | CTTGTCCATAACCTTGATGA |  |
| *MOB2-*5’flank*-*F | AGTAGGCCAATCATTAGGTG | Knock out *MOB2* |
| *MOB2-*5’flank*-*R | CACGGCGCGCCTAGCAGCGGTAGCTAATTAATATAGTTGG |  |
| *MOB2-*3’flank*-*F | GTCAGCGGCCGCATCCCTGCACAAGCTATATGTTGCCAAGAC |  |
| *MOB2-*3’flank*-*R | GGTGTGTTGCAACTGTACTT |  |
| *MOB2-*Check*-*F | TGGGTTCTACTGTTGTCTTG |  |
| *MOB2-*Check*-*R | TTGATTTCACACTGTAGCTT |  |
| *MOB2-*ORF*-*F | TAGCAGAACAGGACAATCTT |  |
| *MOB2-*ORF*-*R | AACAGCTTGACCAGAAGAGT |  |
| *HYM1-*5’flank*-*F | TCTTGTCGTTGCATGGATGG | Knock out *HYM1* |
| *HYM1-*5’flank*-*R | CACGGCGCGCCTAGCAGCGGGCCATAATTATATGTTGTTGA |  |
| *HYM1-*3’flank*-*F | GTCAGCGGCCGCATCCCTGCCGGTTAGTATATATTCTTTA |  |
| *HYM1-*3’flank*-*R | CTAGAGAAAGGTTGCCAAGT |  |
| *HYM1-*Check*-*F | TTAGATCTGACTCGCTCCCA |  |
| *HYM1-*Check*-*R | TGATGATGGCACTAAAGAAT |  |
| *HYM1-*ORF*-*F | TGGTGATGATGAAGTGGAAC |  |
| *HYM1-*ORF*-*R | CTCAATAGAAGCATGGTCAA |  |
| *KIC1-*5’flank*-*F | TATTGGATAGGTGTTGAGTT | Knock out *KIC1* |
| *KIC1-*5’flank*-*R | CACGGCGCGCCTAGCAGCGGTGCATCAACCCACAATAAGA |  |
| *KIC1-*3’flank*-*F | GTCAGCGGCCGCATCCCTGCAATCAAGGATTAGAAGTTCT |  |
| *KIC1-*3’flank*-*R | TCGTTTAGAAGTAGTTATCC |  |
| *KIC1-*Check*-*F | ATTGGCTTTGGTAATTGTGC |  |
| *KIC1-*Check*-*R | GATAATTCTTCTTGTTCTT |  |
| *KIC1-*ORF*-*F | ACTGCCAATTCCACTAAACG |  |
| *KIC1-*ORF*-*R | GTTGTTGCTGTTGATTATGA |  |
| *CAS4-*5’flank*-*F | ATAGAGTATTACTTCTGACC | Knock out *CAS4* |
| *CAS4-*5’flank*-*R | CACGGCGCGCCTAGCAGCGGCTATCATCTTGAGTTATTCT |  |
| *CAS4-*3’flank*-*F | GTCAGCGGCCGCATCCCTGCAAGATATTGTATAGAGATCG |  |
| *CAS4-*3’flank*-*R | GAATACCAAACAAAGCTGCT |  |
| *CAS4-*Check*-*F | TATACTTTATGCTTCGAGG |  |
| *CAS4-*Check*-*R | CAACATTTGGATCATGACTG |  |
| *CAS4-*ORF*-*F | ACTACCTCAAGAATCATCGG |  |
| *CAS4-*ORF*-*R | TGCCAGAGAAGTATTTGTTC |  |
| *SOG2-*5’flank*-*F | TTTATTCAGCCATCAATAG | Knock out *SOG2* |
| *SOG2-*5’flank*-*R | CACGGCGCGCCTAGCAGCGGAACTGACGATCAAACCAAAA |  |
| *SOG2-*3’flank*-*F | GTCAGCGGCCGCATCCCTGCTAAGCATTGTGCGTTAACAG |  |
| *SOG2-*3’flank*-*R | TAACTCTATCTCCAAGGAG |  |
| *SOG2-*Check*-*F | AACTGGAGATTAATTTCTA |  |
| *SOG2-*Check*-*R | GAAATTGCTTCAGCTAGATA |  |
| *SOG2-*ORF*-*F | ATCAGACGAAAACAGTGACG |  |
| *SOG2-*ORF*-*R | GATTTCAAGATTCTCCACT |  |
| *CST20-*ORF*-*F | AGATTCATATTCTCCTGGCAC | Verify *CST20* ORF |
| *CST20-*ORF*-*R | ACTGTAGAAGTCGATGTTCC |  |
| *STE11-*ORF*-*F | TGTCGGTAATAGTGCTTCCAG | Verify *STE11* ORF |
| *STE11-*ORF*-*R | TCTCAAGGTCGATCTATGTGC |  |
| *HST7-*ORF*-*F | TACCCACTGCATCTTCATCTG | Verify *HST7* ORF |
| *HST7-*ORF*-*R | TGAATTTGTGGTTCCAACGGAG |  |
| *CEK1-*ORF*-*F | TCGTCAAGTTTCATTCAACG | Verify *CEK1* ORF |
| *CEK1-*ORF*-*R | TGTAGCTACGTATTCTGTCATG |  |
| *CEK2-*ORF*-*F | ATACATGCCTCTGGATTTGC | Verify *CEK2* ORF |
| *CEK2-*ORF*-*R | ACTTTGTTGGTAGAGATCTG |  |
| *CPH1-*ORF*-*F | ACACATGGAAACACAGAAGAG | Verify *CPH1* ORF |
| *CPH1-*ORF*-*R | TGGTTTGATGTTGTTGCAGTC |  |
| Marker-F | CCGCTGCTAGGCGCGCCGTGACCAGTGTGATGGATATCTGC | Amplify *HIS1, LEU2, URA3, ARG4* for Fusion PCR assays |
| Marker-R | GCAGGGATGCGGCCGCTGACAGCTCGGATCCACTAGTAACG |  |
