# Supplementary material for: Genetic regulation of the development of mating projections in Candida albicans
Source: Emerg Microbes Infect. 2020 Feb 21;9(1):413–26. doi: 10.1080/22221751.2020.1729067 (PMC7048184; doi:10.1080/22221751.2020.1729067)
Supplement: Supplemental Material [file TEMI_A_1729067_SM6265.zip › Table S2. Strains used in this study-2020.1.docx]

**Table S2. Strains used in this study**

| **Strain name** | **Parent strain** | **Genotype** | **Reference** |
| --- | --- | --- | --- |
| SC5314**a** | SC5314 | *MTL***a**/*mtl*α::*FRT* | This study |
| CAI4**a** | CAI4 | *MTL***a**/*mtl*α::*FRT* | This study |
| SN152**a** | SN152 | *MTL***a/***mtlα*::*FRT*,*arg4::hisG/arg4::hisG,his1::hisG /his1::hisG,leu2::hisG/leu2::hisG* | This study |
| SN152*α* | SN152 | *mtl*a::*FRT/MTLα*,*arg4::hisG/arg4::hisG,his1::hisG/his1::hisG,leu2::hisG/leu2::hisG* | This study |
| BWP17**a** | CAI4 | *ura3*::*imm434/ura3::imm434*,*his1::hisG/his1::hisG::CaHIS1,arg4::hisG/arg4::hisG::CaARG4*,*mtl*a:: *FRT /MTLα* | This study |
| GH1349 | WO-1 | *MTLα/α,ura3::FRT/ura3::FRT,arg4::dpl200/arg4:: dpl200-URA3-dpl200* | [1] |
| GH1013 | BWP17 | *MTL***a/a,***ura3::imm434/ura3::imm434,his1::hisG/his1::hisG, arg4::hisG/arg4::hisG* | [1] |
| WT(**a/-**),*MFA1-GFP* | SZ306 | *MF*α*1/MF*α*1::mf*α*1p-GFP* | [2] |
| SN152**a**+p*TET1*- *MTL*α*1* | SN152 | As SN152**a**,but*ADH1/adh1::PTET1-MTLa1-SAT1* | This study |
| SN152**a**+p*TET1* | SN152 | As SN152**a**,but*ADH1/adh1::PTET1-SAT1* | This study |
| WT(**a/a**)+p*TET1*- *MTL*α*1* | GH1013 | As GH1013*,* but*ADH1/adh1::PTET1-MTLa1-SAT1* | This study |
| WT(**a/a**)+ p*TET1* | GH1013 | As GH1013*,*but *ADH1/adh1::PTET1-SAT1* | This study |
| *mfα1*/*mfα1*(**a/**-) | SN152**a** | As SN152**a**,*mfα1::ARG4/mfα1::HIS1* | This study |
| *mfα1/mfα1*(**a**/-)*+* p*TET1-MTLα*1 | SN152**a** | As SN152**a**,*mfα1::ARG4/mfα1::HIS1,ADH1/adh1::PTET1-MTLα1-SAT1* | This study |
| *mfα1/mfα1*(**a**/-)*+* p*TET1* | SN152**a** | As SN152**a**,*mfα1::ARG4/mfα1::HIS1,ADH1/adh1::PTET1-SAT1* | This study |
| *ste2*/ *ste2* (**a/**-) | SN152**a** | As SN152**a**,*ste2::ARG4/ ste2::HIS1* | This study |
| *ste2/ste2*(**a**/-)*+* p*TET1-MTLα*1 | SN152**a** | As SN152**a**,*ste2::ARG4/ste2::HIS1,ADH1/adh1::PTET1-MTLα1-SAT1* | This study |
| *ste2/ste2*(**a**/-)*+* p*TET1* | SN152**a** | As SN152**a**,*ste2::ARG4/ste2::HIS1,ADH1/adh1::PTET1-SAT1* | This study |
| *cph1/ cph1*(**a**/-) | CAI4 | *MTLa/ mtlα*::*FRT* | [2] |
| *cph1/cph1*(**a**/-)*+* p*TET1-MTLα*1 | CAI4 | As*cph1/cph1*(**a**/**-**), but*ADH1/adh1::PTET1-MTLa1-SAT1* | This study |
| *cph1/cph1*(**a**/-)*+* p*TET1* | CAI4 | As*cph1/cph1*(**a**/**-**), but*ADH1/adh1::PTET1-SAT1* | This study |
| *hst7/hst7*(**a**/-) | CAI4 | *MTLa/mtlα*::*FRT* | [2] |
| *hst7/hst7*(**a**/-)*+*p*TET1-MTLα*1 | CAI4 | As*hst7/hst7*(**a**/**-**), but *ADH1/adh1::PTET1-MTLα1-SAT1* | This study |
| *hst7/hst7*(**a**/-)*+* p*TET1* | CAI4 | As*hst7/hst7*(**a**/**-**), but *ADH1/adh1::PTET1- SAT1* | This study |
| *ste11/ste11*(**a**/-) | SC5314 | *MTLa/ mtlα*::*FRT* | [2] |
| *ste11/ste11*(**a**/-)*+PTET1-MTLα*1 | SC5314 | As*ste11/ste11*(**a**/**-**)*, but ADH1/adh1::PTET1-MTLα1-SAT1* | This study |
| *ste11/ste11*(**a**/-)*+PTET1* | SC5314 | As*ste11/ste11*(**a**/**-**)*, but ADH1/adh1::PTET1-SAT1* | This study |
| *cst20/cst20*(**a**/-) | CAI4 | *MTLa/ mtlα*::*FRT* | [2] |
| *cst20/cst20*(**a**/-)*+PTET1-MTLα*1 | CAI4 | As*cst20/cst20*(**a**/**-**)*, but ADH1/adh1::PTET1-MTLα1-SAT1* | This study |
| *cst20/cst20*(**a**/-)*+PTET1* | CAI4 | As*cst20/cst20*(**a**/**-**)*, but ADH1/adh1::PTET1-SAT1* | This study |
| *cek1/cek1, cek2/ cek2* (**a**/-) | CAI4 | *MTLa/ mtlα*::*FRT* | [2] |
| *cek1/cek1, cek2/ cek2*(**a**/-)+ *PTET1-MTLα*1 | CAI4 | As*cek1/cek1, cek2/cek2*(**a**/-)*, but ADH1/adh1::PTET1-MTLα1-SAT1* | This study |
| *cek1/cek1, cek2/ cek2*(**a**/-)+ *PTET1* | CAI4 | As*cek1/cek1, cek2/cek2*(**a**/-)*, but ADH1/adh1::PTET1-SAT1* | This study |
| *ume6/um6*(**a**/**-**) | SN152**a** | As SN152a,but *ume6::ARG4/ ume6::HIS1* | This study |
| *ume6/um6*(**a**/**-**)*+* p*TET1-MTLα*1 | SN152**a** | As*ume6/um6*(**a**/**-**)*,ADH1/adh1::PTET1-MTLα1-SAT1* | This study |
| *ume6/um6*(**a**/**-**)*+* p*TET1* | SN152**a** | As*ume6/um6*(**a**/**-**)*,ADH1/adh1::PTET1-SAT1* | This study |
| *tec1/tec1*(**a**/**-**) | SN152**a** | As SN152a,but *tec1::ARG4/tec1::HIS1* | This study |
| *tec1/tec1*(**a**/**-**)*+* p*TET1-MTLα*1 | SN152**a** | As*cek1/cek1, cek2/cek2*(**a**/-)*, but ADH1/adh1::PTET1-MTLα1-SAT1* | This study |
| *tec1/tec1*(**a**/**-**)*+* p*TET1* | SN152**a** | As*cek1/cek1, cek2/cek2*(**a**/-)*, but ADH1/adh1::PTET1-SAT1* | This study |
| *hgc1/hgc1*(**a**/**-**) | BWP17**a** | As BWP17a,*hgc1::FRT/hgc1::FRT* | This study |
| *hgc1/hgc1*(**a**/**-**)*+* p*TET1-MTLα*1 | BWP17**a** | *hgc1::FRT/hgc1::FRT,ADH1/adh1::PTET1-MTLα1 -SAT1* | This study |
| *hgc1/hgc1*(**a**/**-**)*+* p*TET1* | BWP17**a** | *hgc1::FRT/hgc1::FRT,ADH1/adh1::PTET1-SAT1* | This study |
| *cbk1/ cbk1* (**a**/**-**) | SN152**a** | As SN152a,but *cbk1::ARG4/ cbk1::HIS1* | This study |
| *cbk1/cbk1*(**a**/**-**)*+PTET1-MTLα*1 | SN152**a** | *cbk1::ARG4/cbk1::HIS1,ADH1/adh1::PTET1-MTLα1 -SAT1* | This study |
| *cbk1/cbk1*(**a**/**-**)*+PTET1* | SN152**a** | *cbk1::ARG4/cbk1::HIS1,ADH1/adh1::PTET1-SAT1* | This study |
| *cbk1/cbk1*(**a**/**-**)+ p*ACT1-WOR1* | SN152**a** | *cbk1::ARG4/cbk1::HIS1,ADE2/ade2::ACT1p-WOR1-URA3-SAT1* | This study |
| *cbk1/cbk1*(-/α)+ p*ACT1-WOR1* | SN152α | *cbk1::ARG4/cbk1::LEU3,ADE2/ade2::ACT1p-WOR1-URA3-SAT1* | This study |
| *WT*(**a**/**-**)+ p*ACT1-WOR1* | SN152a | As SN152 a,*but arg4::hisG::ARG4/arg4::hisG*  *his1::hisG::HIS1/his1::hisG*,*ADE2/ade2::ACT1p-WOR1-URA3-SAT1* | This study |
| *WT*(-/α)+ p*ACT1-WOR1* | SN152α | As SN152α,*but arg4::hisG::ARG4/arg4::hisG*  *his1::hisG::HIS1/his1::hisG*,*ADE2/ade2::ACT1p-WOR1-URA3-SAT1* | This study |
| *mob2/ mob2* (**a**/**-**) | SN152**a** | As SN152a,but *mob2::ARG4/ mob2::HIS1* | This study |
| *mob2/mob2*(**a**/**-**)+ p*ACT1-WOR1* | SN152**a** | *As mob2/mob2*(**a**/**-**)*,ADE2/ade2::ACT1p-WOR1-URA3-SAT1* | This study |
| *hym1/ hym1* (**a**/**-**) | SN152**a** | As SN152a,but *hym1::ARG4/ hym1::HIS1* | This study |
| *hym1/hym1*(**a**/**-**)+ p*ACT1-WOR1* | SN152**a** | *hym1::ARG4/hym1::HIS1,ADE2/ade2::ACT1p-WOR1-URA3-SAT1* | This study |
| *kic1/ kic1* (**a**/**-**) | SN152**a** | As SN152a,but *kic1::ARG4/ kic1::HIS1* | This study |
| *kic1/kic1*(**a**/**-**)+ p*ACT1-WOR1* | SN152**a** | *kic1::ARG4/kic1::HIS1,ADE2/ade2::ACT1p-WOR1-URA3-SAT1* | This study |
| *cas4/ cas4* (**a**/**-**) | SN152**a** | As SN152a,but *cas4::ARG4/ cas4::HIS1* | This study |
| *cas4/cas4*(**a**/**-**)+ p*ACT1-WOR1* | SN152**a** | *cas4::ARG4/cas4::HIS1,ADE2/ade2::ACT1p-WOR1-URA3-SAT1* | This study |
| *sog2/ sog2* (**a**/**-**) | SN152**a** | As SN152a,but *sog2::ARG4/ sog2::HIS1* | This study |
| *sog2/sog2*(**a**/**-**)+ p*ACT1-WOR1* | SN152**a** | *sog2::ARG4/sog2::HIS1,ADE2/ade2::ACT1p-WOR1-URA3-SAT1* | This study |
| *mob2/mob2*(**a**/**-**)*+*p*TET1-MTLα*1 | SN152**a** | *mob2::ARG4/mob2::HIS1,ADH1/adh1::PTET1-MTLα1-SAT1* | This study |
| *mob2/mob2*(**a**/**-**)*+*p*TET1* | SN152**a** | *mob2::ARG4/mob2::HIS1,ADH1/adh1::PTET1-SAT1* | This study |
| *hym1/hym1*(**a**/**-**)*+*p*TET1-MTLα*1 | SN152**a** | *hym1::ARG4/hym1::HIS1,ADH1/adh1::PTET1-MTLα1-SAT1* | This study |
| *hym1/hym1*(**a**/**-**)*+*p*TET1* | SN152**a** | *hym1::ARG4/hym1::HIS1,ADH1/adh1::PTET1-SAT1* | This study |
| *kic1/kic1*(**a**/**-**)*+*p*TET1-MTLα*1 | SN152**a** | *kic1::ARG4/kic1::HIS1,ADH1/adh1::PTET1-MTLα1-SAT1* | This study |
| *kic1/kic1*(**a**/**-**)*+*p*TET1* | SN152**a** | *kic1::ARG4/ kic1::HIS1,ADH1/adh1::PTET1-SAT1* | This study |
| *cas4/cas4*(**a**/**-**)*+*p*TET1-MTLα*1 | SN152**a** | *cas4::ARG4/cas4::HIS1,ADH1/adh1::PTET1-MTLα1-SAT1* | This study |
| *cas4/cas4*(**a**/**-**)*+*p*TET1* | SN152**a** | *cas4::ARG4/cas4::HIS1,ADH1/adh1::PTET1-SAT1* | This study |
| *sog2/sog2*(**a**/**-**)*+*  p*TET1-MTLα*1 | SN152**a** | *sog2::ARG4/sog2::HIS1,ADH1/adh1::PTET1-MTLα1-SAT1* | This study |
| *sog2/sog2*(**a**/**-**)*+*p*TET1* | SN152**a** | *sog2::ARG4/sog2::HIS1,ADH1/adh1::PTET1-SAT1* | This study |
| *mob2/ mob2* (α/-) | SN152α | As SN152α,but *mob2::ARG4/ mob2::HIS1* | This study |
| *hym1/ hym1* (α/-) | SN152α | As SN152α,but *hym1::ARG4/ hym1::HIS1* | This study |
| *kic1/ kic1* (α/-) | SN152α | As SN152α,but *kic1::ARG4/ kic1::HIS1* | This study |
| *cas4/ cas4* (α/-) | SN152α | As SN152α,but *cas4::ARG4/ cas4::HIS1* | This study |
| *sog2/ sog2* (α/-) | SN152α | As SN152α,but *sog2::ARG4/ sog2::HIS1* | This study |
| *mob2/mob2*(α/-)+ p*ACT1-WOR1* | SN152α | *As mob2/mob2*(α/-)*,ADE2/ade2::ACT1p-WOR1-URA3-SAT1* | This study |
| *hym1/hym1*(α/-)+ p*ACT1-WOR1* | SN152α | *hym1::ARG4/hym1::HIS1,ADE2/ade2::ACT1p-WOR1-URA3-SAT1* | This study |
| *kic1/kic1*(α/-)+ p*ACT1-WOR1* | SN152α | *kic1::ARG4/kic1::HIS1,ADE2/ade2::ACT1p-WOR1-URA3-SAT1* | This study |
| *sog2/sog2*(α/-)+ p*ACT1-WOR1* | SN152α | *sog2::ARG4/sog2::HIS1,ADE2/ade2::ACT1p-WOR1-URA3-SAT1* | This study |
| *cas4/cas4*(α/-)+ p*ACT1-WOR1* | SN152α | *cas4::ARG4/cas4::HIS1,ADE2/ade2::ACT1p-WOR1-URA3-SAT1* | This study |
| *ras1/ras1*(**a**/**a**) | GH1013 | As GH1013, but *ras1::ARG4/ras1::URA3* | This study |
| *Cyr1/cyr1*(**a**/**a**) | GH1013 | As GH1013, but *cyr1::ARG4/cyr1::HIS1* | [3] |
| *efg1/efg1*(**a**/**a**) | CAI4 | *MTL***a**/*mtlα1::HisGmtlα2::HisG,ura3::imm434/ura3::imm434, efg1::dpl200/efg1::URA3-dpl200* | [3] |
| *flo8/flo8*(**a**/**a**) | CCF3 | *MTL***a/a***, ura3:: imm434/ura3:: imm434flo8::hisG/flo8::hisG-URA3-hisG* | [3] |
| *flo8/flo8*(**a**/**a**)+ p*ACT1-WOR1* | CCF3 | *As flo8/flo8*(**a**/**a**), but *ADE2/ade2::ACT1p-WOR1-URA3-SAT1* | This study |
| CAI4**a**+ p*ACT1-WOR1* | CAI4**a** | *As* CAI4**a,** but *ADE2/ade2::ACT1p-WOR1-URA3-SAT1* | This study |
| *tpk1/tpk1*(**a**/-) | SN152 | *tpk1/tpk1, MTL***a**/*mtα::FRT* | [4] |
| *tpk2/tpk2*(**a**/-) | SN152 | *tpk2/tpk2, MTL***a**/*mtα::FRT* | [4] |
| *tpk1/tpk1, tpk2/tpk2*(**a**/-) | SN152 | *tpk1/tpk1, tpk2/tpk2, MTL***a**/*mtα::FRT* | [4] |
| *tpk1/tpk1, tpk2/tpk2*(α/-) | SN152 | *tpk1/tpk1, tpk2/tpk2, mtl*a::*FRT/MTLα* | [4] |

**Reference:**

1. Huang G, Srikantha T, Sahni N, et al. CO(2) regulates white-to-opaque switching in *Candida albicans*. Current Biology, 2009, 19: 330-334.
2. Tao L, Cao C, Liang W, et al. White cells facilitate opposite-and same-sex mating of opaque cells in *Candida albicans*. PLoS genetics, 2014, 10(10): e1004737.
3. Guan G, Xie J, Tao L, et al. Bcr1 plays a central role in the regulation of opaque cell filamentation in *Candida albicans.* Molecular microbiology, 2013, 89(4): 732-750.
4. Cao C, Wu M, Bing J, et al. Global regulatory roles of the c AMP/PKA pathway revealed by phenotypic, transcriptomic and phosphoproteomic analyses in a null mutant of the PKA catalytic subunit in *Candida albicans*. Molecular microbiology, 2017, 105(1): 46-64.
