# Supplementary figures and images for: Genetic regulation of the development of mating projections in Candida albicans
Source: Emerg Microbes Infect. 2020 Feb 21;9(1):413–26. doi: 10.1080/22221751.2020.1729067 (PMC7048184; doi:10.1080/22221751.2020.1729067)

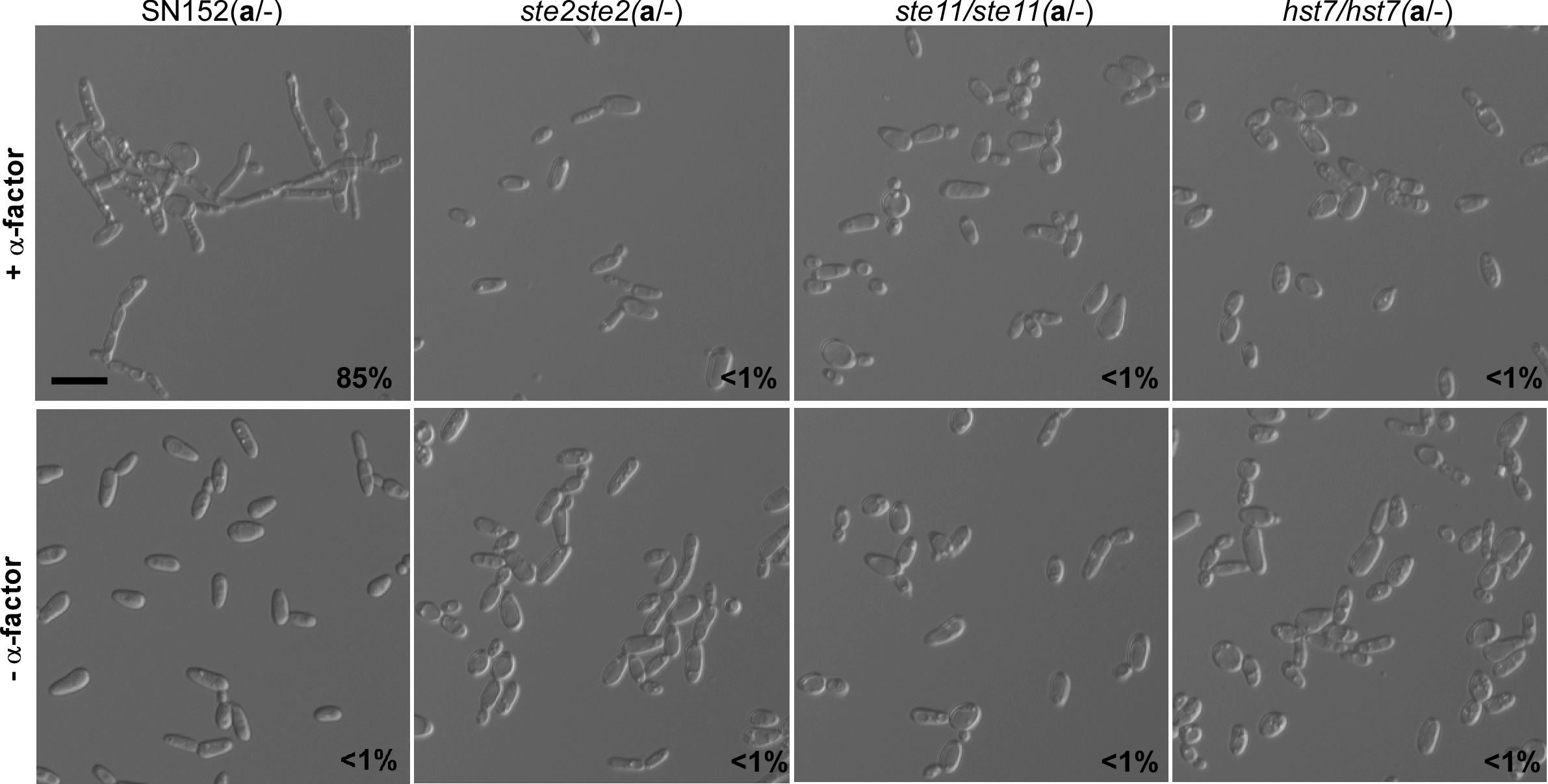

Supplement: Supplemental Material [file TEMI_A_1729067_SM6265.zip › FIGURE S1-20200203.jpg]

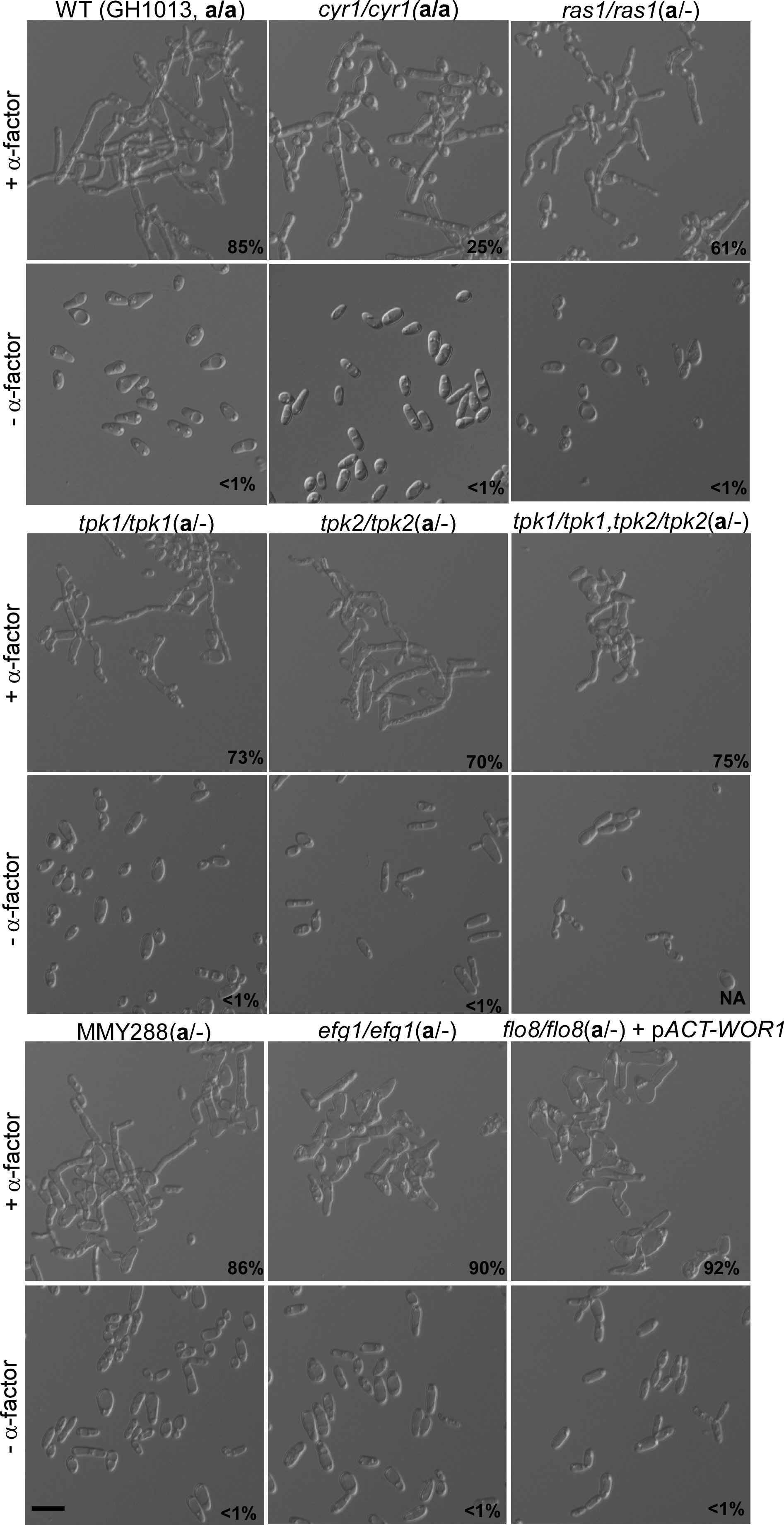

Supplement: Supplemental Material [file TEMI_A_1729067_SM6265.zip › FIGURE S2-20200203.jpg]

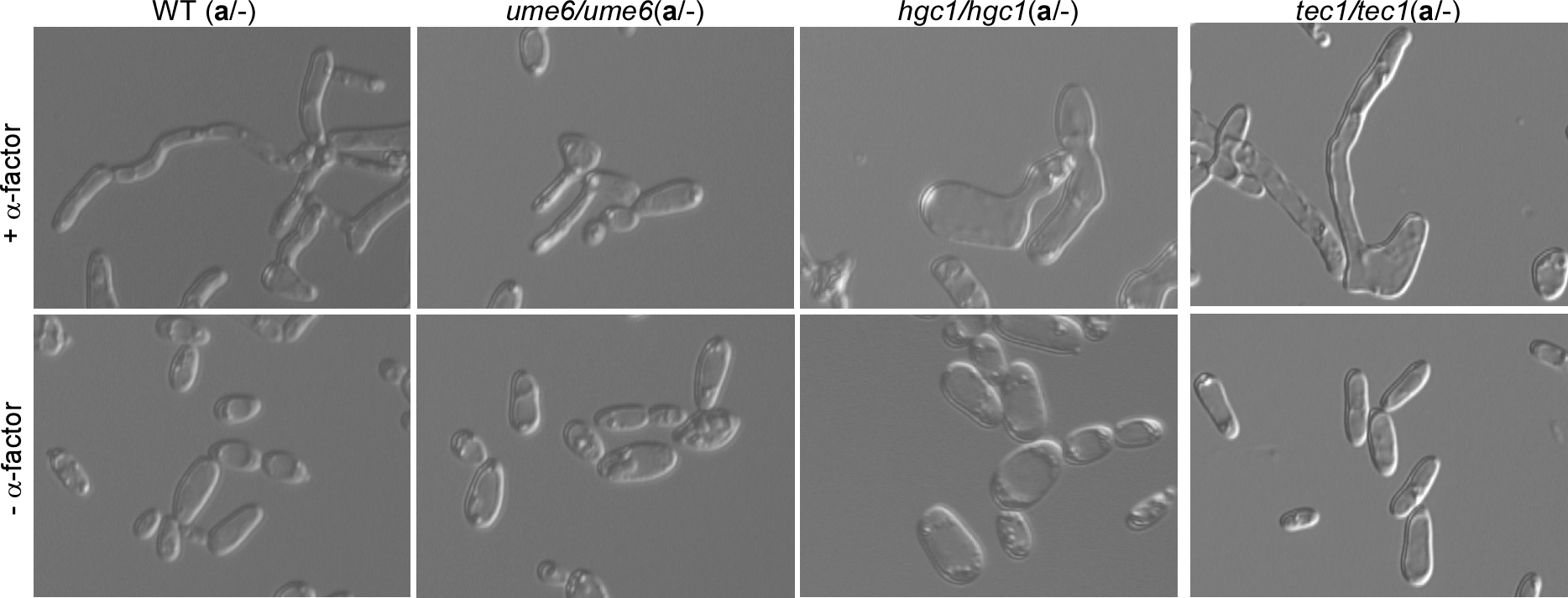

Supplement: Supplemental Material [file TEMI_A_1729067_SM6265.zip › FIGURE S3-0805.tif]

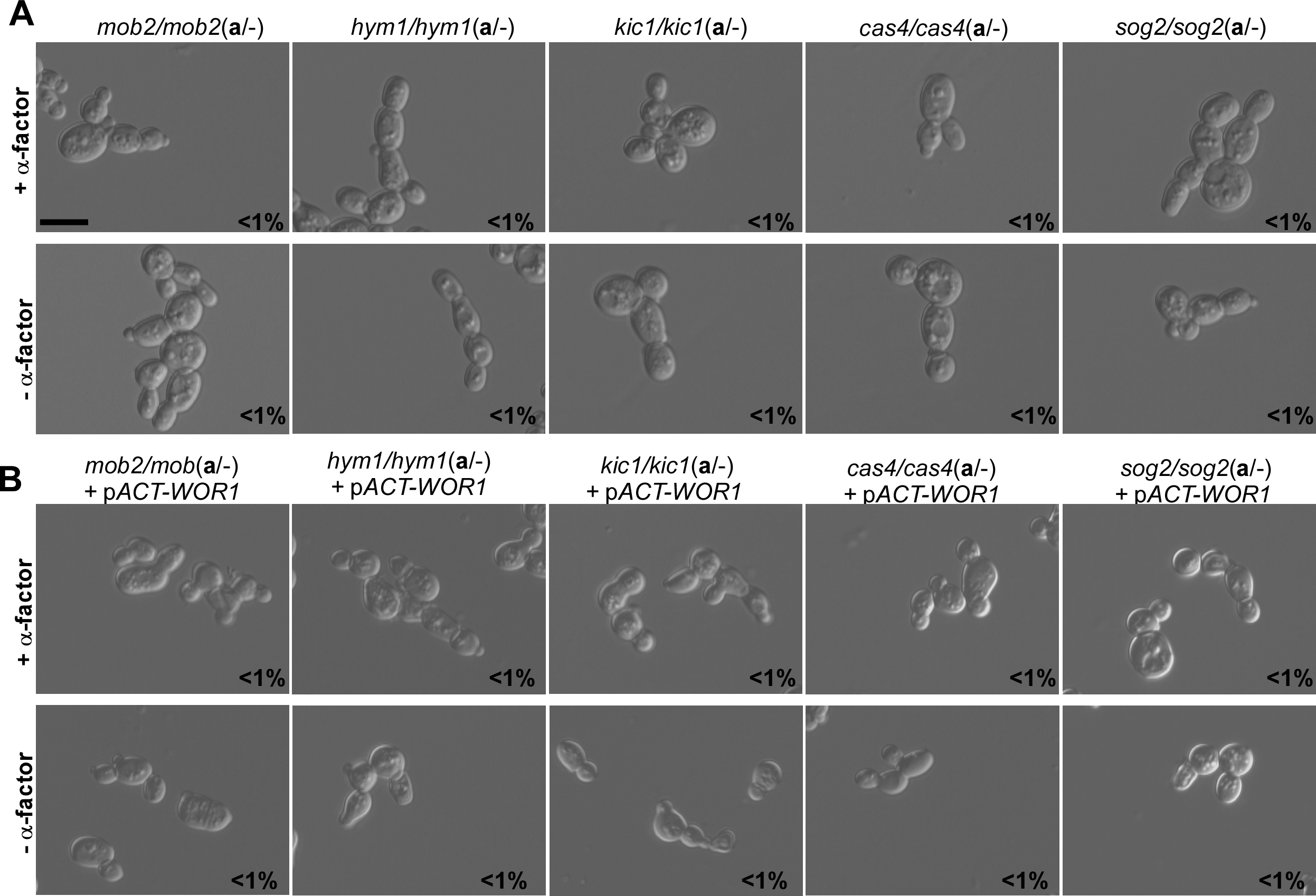

Supplement: Supplemental Material [file TEMI_A_1729067_SM6265.zip › FIGURE S4-20200203.jpg]

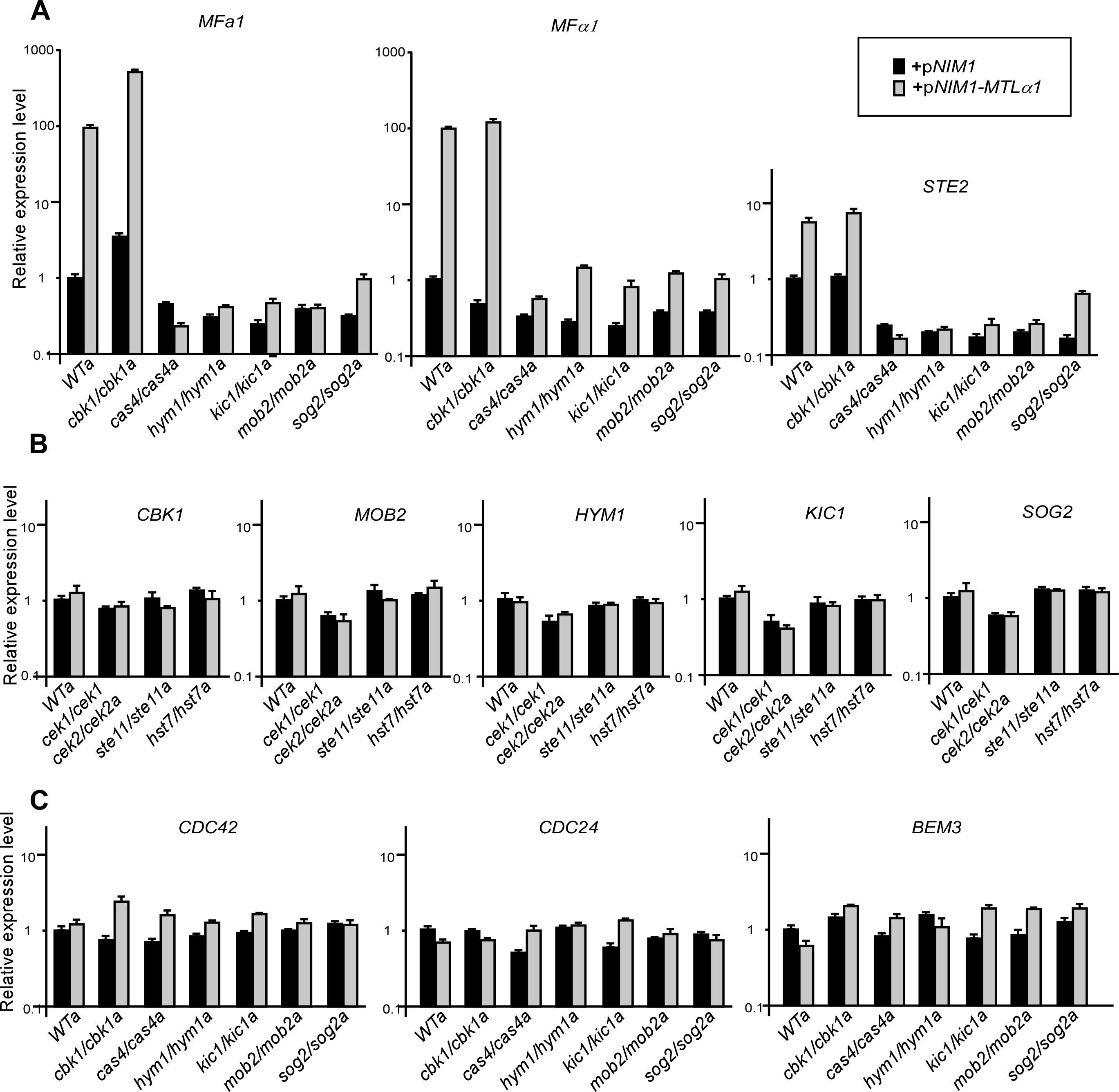

Supplement: Supplemental Material [file TEMI_A_1729067_SM6265.zip › Figure S5-012020.tif]
